# Hydrogen Bond Donors in the Catalytic Pocket: The Case of the Ring-Opening Polymerization of Cyclic Esters Catalyzed by an Amino-Propoxide Aluminum Complex

**DOI:** 10.3390/polym16213047

**Published:** 2024-10-30

**Authors:** Salvatore Impemba, Antonella Viceconte, Irene Tozio, Shoaib Anwar, Gabriele Manca, Stefano Milione

**Affiliations:** 1 Department of Chemistry and Biology, University of Salerno, Via Giovanni Piano II, 84084 Fisciano, Salerno, Italy; simpemba@unisa.it (S.I.); sanwar@unisa.it (S.A.); 2 CNR-ICCOM, Consiglio Nazionale delle Ricerche, Via Madonna del Piano 10, 50019 Sesto Fiorentino, Firenze, Italy

**Keywords:** ring-opening polymerization, hydrogen bond, polyesters, aluminum, density functional theory

## Abstract

A new aluminum complex (NSO)AlMe_2_ featuring a hydrogen bond donor on the ligand backbone has been synthesized via the reaction of AlMe_3_ with 1-((2-(isopropylamino)phenyl)thio)propan-2-ol (NSO-H) and spectroscopically characterized. In the complex, the aluminum atom is in a distorted tetrahedral coordination sphere determined by the anionic oxygen and neutral nitrogen atoms of the ligand and by the two carbon atoms of the alkyl groups. After proper activation, the complex (NSO)AlMe_2_ was able to promote the ring-opening polymerization of *L*-, *rac*-lactide, *ε*-caprolactone and *rac*-*β*-butyrolactone. The polymerization of *rac*-lactide was faster than that of *L*-lactide: in a toluene solution at 80 °C, the high monomer conversion of 100 equivalents was achieved in 1.5 h, reaching a turnover frequency of 63 mol_LA_·mol_Al_^–1^·h^–1^. The experimental molecular weights of the obtained polymers were close to those calculated, assuming the growth of one polymer chain for one added alcohol equivalent and the polydispersity indexes were monomodal and narrow. The kinetic investigation of the polymerization led to the determination of the apparent propagation constants and the Gibbs free energies of activation for the reaction; the terminal groups of the polymers were also identified. The complex (NSO)AlMe_2_ was active in harsh conditions such as at a very low concentration or in the melt using technical-grade *rac*-lactide. A relatively high level of activity was observed in the ring-opening polymerization of *ε*-caprolactone and *rac*-*β*-butyrolactone. DFT calculations were performed and revealed the central role of the NH function of the coordinated ligand. Acting as a hydrogen bond donor, it docks the monomer in the proximity of the metal center and activates it toward the nucleophilic attack of the growing polymer chain.

## 1. Introduction

Plastics are one of the major inventions of the 20th century. Thanks to their properties, easy processibility and the relatively low cost, plastics have found applications in a significant and ever-expanding range of products. Their relevance is witnessed by their continuous increase in production that reached 400.3 million metric tons in 2022 [1]. However, plastic derived by petrochemicals has raised severe environmental issues and the academic research has addressed the development of green and degradable alternatives [2,3]. Among the most representative examples of environmentally relevant polymeric materials are the aliphatic polyesters [4,5,6]. They can be efficiently synthesized by the ring-opening polymerization (ROP) of the cyclic esters; the catalysis of the reaction by means of coordination metal complexes provides polymers with controlled properties and avoids the formation of small-molecule byproducts [7,8,9]. The metal center and the coordination sphere determined by the ancillary ligand play a pivotal role in the catalytic performance of the complexes. The research interest is mainly aimed at the development of catalytic systems based on cheap and non-toxic metals, such as Fe [10,11,12], Zn [13,14], Zr, Hf [15,16,17,18] and alkaline/alkaline earth [19,20] metals able to promote the ROP of several cyclic esters. Aluminum, due to its earth-abundance, relatively low toxicity and low cost is of particular interest for developing sustainable catalysis with non-endangered metals. Numerous Al complexes have been reported [21,22,23]; relevant examples are those that found applications in the stereoselective polymerization of *rac*-lactide (*rac*-LA). The first report was by Spassky et al., in which an isotactic polylactide (PLA) with an enhanced melting temperature was obtained by using a chiral binaphthyl Schiff bases (*salen*-type) [24,25]. Nomura and co-workers, using an Al-achiral ligand complex composed by two salicylaldimine units linked by a propylene bridge, obtained a highly isotactic multiblock PLA [26,27]. Lamberti et al. succeeded in the preparation of a gradient isotactic multiblock PLA by using an Al complex supported by enantiomerically pure aminomethylpyrrolidine-based *salalen* ligands [28]. However, Al catalysts generally display low activity and require a high temperature or long time for a reaction to reach satisfactory monomer conversions. An exception is a series of highly active Al(III) complexes bearing catechol-amine ligands [29,30]. For example, the Al complex supported by the ligand composed by two ortho-aminophenolate moieties linked by a propyl bridge, when activated with BnOH, led to the full conversion to PLA with a slight isotactic bias within 90 min at room temperature in THF. The series of catechol-amine-based Al(III) complexes are characterized by the presence of NH functions near the metal center that act as hydrogen bond donors. Experimental and theoretical studies have evidenced that the formation of hydrogen bonds between the ligand and the monomer has a beneficial effect on the polymerization activity. Another example is the Al complex reported by Jones et al. that bears a ligand composed of a phenoxyimine and ortho-aminophenolate moieties linked by an ethyl bridge. When activated with benzyl alcohol, this complex was able to convert 100 equivalents of *rac*-LA in 30 min in toluene solution at 80 °C [31].

In the framework of our efforts to develop new coordination environments, we have been interested in exploring the influence of noncovalent interactions on the catalytic performances of Al complexes. Herein, we report the synthesis and the characterization of a new Al complex supported by a ligand featuring a hard alcholate group, a soft thioether group and an additional NH group that has the scope of acting as a hydrogen bond donor. When assessed in the ROP of different cyclic esters (*L*-, *rac*-LA, *ε*-caprolactone (*ε*-CL) and β-butyrolactone (*β*-BL)), the complex revealed good activities. Using density functional theory (DFT) calculations, we showed the pivotal role of the NH function in the reaction path of the ROP.

## 2. Experimental Section

The description of materials, method, instruments, and measurements is provided in the Supporting Information.

### 2.1. Synthesis of 1-((2-(Isopropylamino)phenyl)thio)propan-2-ol (NSO-H)

*Step 1. Synthesis of 1-(2-amino-phenyl)thio)propan-2-ol*

Propylene oxide (4.36, 75 mmol) was added dropwise to a solution of 2-aminothiophenol (8.55, 68 mmol) and sodium hydroxide (2.73, 68 mmol) in methanol (250 mL) at room temperature. The reaction mixture solution was refluxed for 2 h. After that, the solvent was removed under vacuum, water (50 mL) was added and the reaction mixture was extracted with diethyl ether (4 × 50 mL). The organic layer was dried with anhydrous MgSO_4_, filtered and evaporated to dryness to get a brown oil. Yield 9.91 g, 79%. ^1^H NMR (400.13 MHz, CD_2_Cl_2_, 25 °C): δ 6.67–7.40 (m, 4H, ArH), 4.43 (s, 2H, NH_2_), 3.72 (m, 1H, O-CH), 2.60–2.92 (m, 2H, S-CH_2_), 1.17 (d, *J* = 6.20 Hz, 3H, CH_3_). ^13^C NMR (100.62 MHz, CD_2_Cl_2_, 25 °C): δ 22.10, 44.73, 66.32, 115.49, 117.70, 119.09, 130.36, 136.51, 148.91.

*Step 2. Synthesis of 1-((2-(isopropylamino)phenyl)thio)propan-2-ol*

1-(2-amino-phenyl)thio)propan-2-ol (9.8 g, 53 mmol), zinc (34.8, 0.53 mol), acetic acid (250 mL) and acetone (30.9 g, 0.53 mol) were added to a 500 mL round-bottom one-necked flask equipped with a condenser and Teflon-sealed stir bar. The mixture was stirred to room temperature for 24 h. After that, it was quenched with a 30% NH_3_ aqueous solution (350 mL) and dichloromethane (300 mL). The organic layer was washed with water (3 × 100 mL) and dried with anhydrous MgSO_4_. After that, the solution was concentrated under reduced pressure, and the product was purified by column chromatography on 70–230 mesh silica gel to afford a pale yellow oil. Yield: 11.2 g, 94%. ^1^H NMR (400.13 MHz, C_2_D_2_Cl_4_, 25 °C): δ 6.62–7.45 (m, 4H, ArH), 4.92 (s, 1H, NH), 3.63–3.77 (m, 2H, O-CH; N-CH), 2.61–2.87 (m, 2H, S-CH_2_), 1.27 (d, *J* = 6.27 Hz, 3H, CH_3_), 1.22 (d, *J* = 6.10 Hz, 3H, CH_3_). ^13^C NMR (100.62 MHz, C_2_D_2_Cl_4_, 25 °C): δ 21.84, 22.74, 22.83, 43.89, 44.22, 65.96, 110.98, 116.56, 116.61, 130.38, 136.48, 148.20. HRMS (MALDI). Calcd for C_12_H_20_NOS ([M + H]^+^): *m*/*z* 226.13. Found: *m*/*z* 226.13.

### 2.2. Synthesis of (NSO)AlMe_2_ Complex

AlMe_3_ (0.17 g, 2.38 mmol) was added to a stirred solution of (NSO-H) (0.53 g, 2.35 mmol) in benzene (3 mL) at room temperature. The resulting solution was refluxed for 12 h, after which the volatiles were removed under vacuum. The crude product was washed with hexane (5 mL) to give the complex as a yellow oil (0.48 g, 72%). ^1^H NMR (400.13 MHz, C_2_D_2_Cl_4_, 25 °C): δ −0.81 (d, *J* = 1.56 Hz, 6H, Al-CH_3_), 1.25 (d, *J* = 6.17 Hz, 6H, CH_3_), 1.37 (d, *J* = 6.00 Hz, 3H, CH_3_), 2.64 (dd, *J*_1_ = 13.01 Hz, *J*_2_ = 7.49 Hz, 1H, S-CH), 2.93 (dd, *J*_1_ = 12.91 Hz, *J*_2_ = 4.73 Hz, 1H, S-CH), 3.66 (m, 1H, N-CH), 4.00 (m, 1H, O-CH), 4.76 (d, *J* = 7.97 Hz, 1H, NH), 6.64 (m, 2H, ArH), 7.21 (m, 1H, ArH), 7.36 (m, 1H, ArH). ^13^C NMR (100.62 MHz, C_2_D_2_Cl_4_, 25 °C): δ −8.62, 22.20, 22.80, 22.85, 42.52, 43.87, 69.73, 110.72, 116.22, 116.29, 130.19, 135.62, 147.89. Anal. Calcd for C_14_H_24_AlNOS (281.39 g/mol): C, 59.76; H, 8.60; N, 4.98. Found: C, 59.97; H, 8.78; N, 5.02.

### 2.3. Typical Procedure for Cyclic Esters’ Polymerization

In a glovebox, a Schlenk flask (10 cm^3^) was charged sequentially with the monomer (*rac*- or *L*-lactide, *ε*-caprolactone or *rac*-*β*-butyrolactone), catalyst and solvent (2.0 mL). The mixture was heated thermostatically at the required temperature. At specified time intervals, a small amount of the polymerization mixture was sampled using a pipette and quenched in wet CDCl_3_. This fraction was subjected to monomer conversion determination, which was monitored by the integration of monomer versus polymer methine resonances in the ^1^H NMR spectrum (CDCl_3_) at 25 °C. After the required polymerization time, the reaction mixture was quenched with wet *n*-hexane. The obtained polymer was collected by filtration and dried in vacuum at 40 °C for 16 h. 

### 2.4. Computational Details

All DFT calculations were performed at the GGA level with the Gaussian 09 set of programs [32], using the BP86 functional of Becke and Perdew [33,34]. The electronic configuration of the molecular systems was described with the 6–31G(d) basis set for all the atoms. Geometry optimizations were performed without symmetry constraints, and all the obtained structures were validated as minima or transition states by using vibrational frequency calculations. 

The reported Gibbs free energies have been obtained by adding thermal corrections in gas phase to electronic energy in solvent (CPCM model) [35] computed via single point calculation in toluene at the BP86-D3 level with the triple-ζ basis set of Ahlrichs (TZVP). The atomic charges have been calculated within ATP theory [36]. The buried volume calculations were performed with the SambVca 2.1 package, a software free of charge, developed by Cavallo et al. [37]. The radius of the sphere around the metal center was set to 3.5 Å, while for the atoms, we adopted the Bondi radii scaled by 1.17 and a mesh of 0.1 Å was used to scan the sphere for buried voxels. Non-covalent interaction (NCI) surfaces were created using a script developed by Prof. Henry Rzepa, available at http://doi.org/10.14469/hpc/3660 [38,39,40].

## 3. Results and Discussion

### 3.1. Synthesis and Characterization of (NSO)AlMe_2_ Complex

**Scheme 1:**
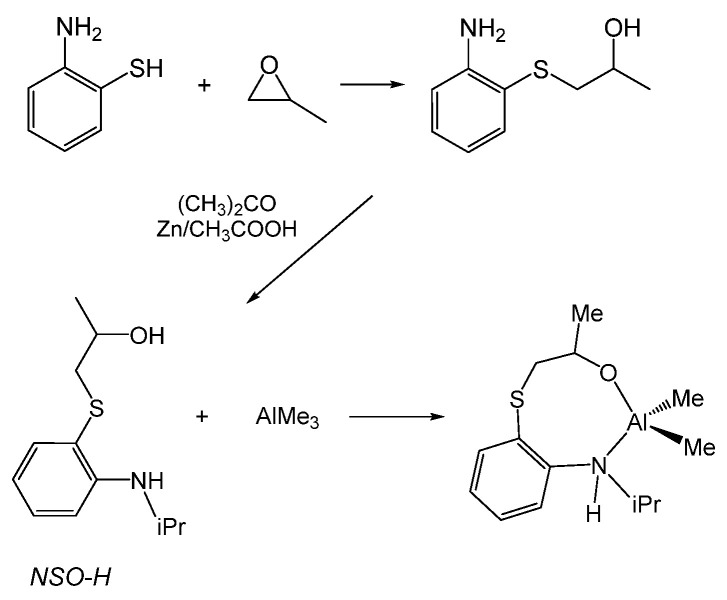
Synthesis of NSO-H and (NSO)AlMe_2_.

The ligand used in this work (NSO-H) features a linear array of an alcoholic group, a thioether group and an amine group; the structure is reported in Scheme 1. It was synthesized by reacting 2-aminothiophenol with propylene oxide in the presence of sodium hydroxide, according to a modified literature procedure [41]. The aniline function was alkylated in the subsequent step by treating the obtained intermediate with acetone in the presence of zinc and acetic acid, according to Scheme 1 [42].

The ligand NSO-H was isolated as a yellow oil in good yield (85%) and was characterized by ^1^H and ^13^C NMR and Mass Spectroscopy. The ^1^H NMR resonances (Figure S1 in the Supporting Information) appear in the intervals of chemical shifts expected for this compound. The methylene protons show the typical pattern displayed by diasterotopic protons caused by the vicinal chiral center. The proton of the NH group appeared as a broad resonance at 4.92 ppm. The reaction with AlMe_3_ was initially studied using NMR spectroscopy. Soon after the mixing of the reagents (at room temperature in C_6_D_6_), the ^1^H NMR spectrum revealed the disappearance of the -OH signal and the permanence of a resonance due to the NH proton, implying that the ligand had reacted with AlMe_3_ to form a monoanionic alcholate derivative. The prolongated heating of the reaction mixture did not change the outcome of the reaction. The integral ratio of the signals due to the methyl groups bound to the Al atom and the methyl group of the ligand were in a mole ratio of 2:1, confirming the stoichiometry of the complex as (NSO)AlMe_2_. With respect to the ligand, the resonances of the -CH_2_CH(CH_3_)- bridge were shifted downfield because of the localization of the partial negative charge on the oxygen atoms. The proton NH appeared as a doublet at 4.76 ppm, suggesting the coordination of the nitrogen atom to the acidic Al center. No element of symmetry was found in the pattern of resonances of the coordinated ligand. Only the methyl groups bound to the Al atom appear as a singlet. These data indicate that the complex is present in the solution in a racemic mixture, with a rapid exchange between two enantiomers.

**Figure 1:**
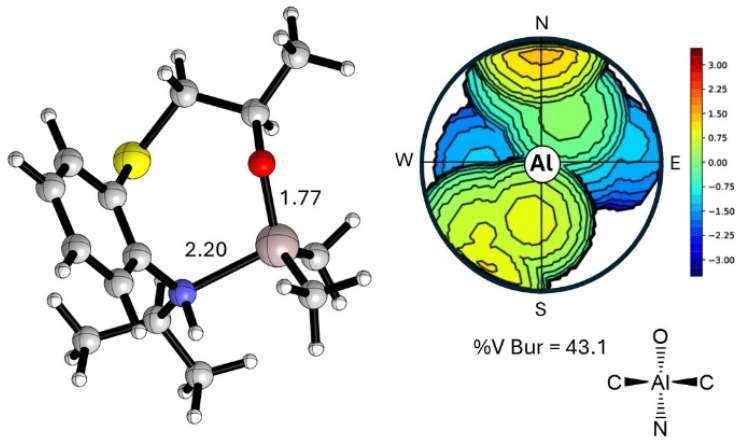
Minimum energy structure and steric encumbrance determined by the ligand for complex (NSO)AlMe_2_. The isocontour curves are given in Å.

The (NSO)AlMe_2_ complex was then synthesized on a preparative scale by the direct treatment of the ligand with AlMe_3_ in a benzene solution (Scheme 1). The complex was isolated in a good yield as pale-yellow low-melting waxes; this impeded the structural characterization by X-ray diffraction studies. With the lack of any hints on the structural arrangement, DFT calculations were undertaken. The obtained minimum energy structure (**1_N_**) is reported in Figure 1, featuring the Al atom in a distorted tetrahedral coordination sphere, determined by the two methyl groups and by the NSO ligand. This last acts as a bidentate ligand binding the metal through the anionic oxygen atom and the neutral nitrogen atom. The bond distances are 1.77 Å and 2.20 Å for the Al-O and Al-N bonds, respectively. In principle, the NSO ligand can coordinate to the Al center using the sulfur atom rather than through the nitrogen atom. This isomer (**1_S_**) was successfully located; the sulfur atom was at 2.60 Å from the Al center with a trigonal pyramidal geometry. Additionally, **1_S_** was less stable than **1_N_** by 5.8 kcal/mol. The difference in energy is probably due to the higher Lewis base character of the amine group compared to the thioether one. A potential isomer, featuring the NSO as a tridentate ligand with the additional coordination of the oxygen, was discharged given the failure of all the computational attempts to optimize it.

**Figure 2:**
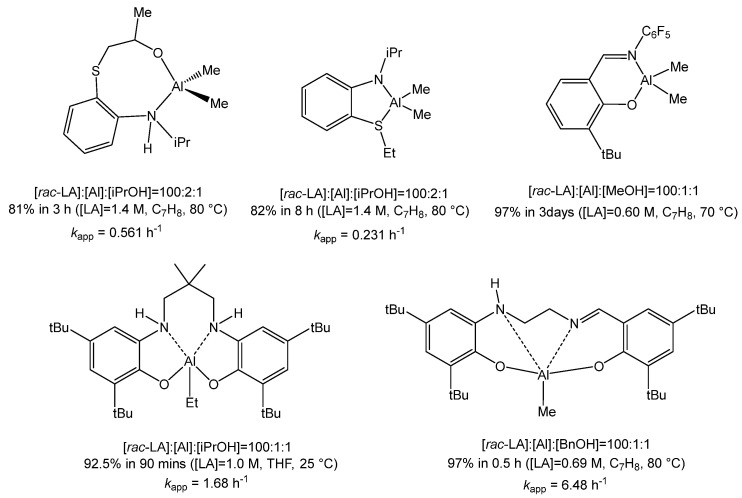
Catalytic performances of selected aluminum complexes.

The Lewis acidity and steric hindrance around the Al metal center are two important factors that may influence the catalytic activity. The Lewis acidity was estimated with the partial charge of the Al atom obtained through natural population analysis (NPA) [36], while steric hindrance due to the coordinated ligands was determined from the percentage of the total volume occupied (%Vbur) by the ligand of the sphere with a radius of 3.5 Å and the metal at the core using the SambVca package, a software free of charge, developed by Cavallo et al. [37]. The partial charge at the Al atom is 1.83 au. This value is close to that found for other monomeric Al complexes featuring bidentate monoanionic ligands such as phenoxy-imine or phenoxy-thioether. The steric map (Figure 2) shows that the oxygen and the nitrogen atoms hamper the apical and one of the equatorial positions of the tetrahedron at the center of which is the Al atom. The volume occupied by the ligand is only 43.1%.

### 3.2. Polymerization Results

The ability of the (NSO)AlMe_2_ complex to promote the ROP of *rac*- or *L*-LA has been investigated under different experimental conditions. Initially, the polymerizations were carried out in a toluene solution at 80 °C. The representative results are reported in Table 1. As the methyl groups and the NSO ligand are both poorly starting groups, in the absence of a co-initiator, the complex was inactive. To achieve an efficient catalyst, isopropyl alcohol (two equivalents) was added in situ. In these conditions, the complex resulted as active: in the ROP of *L*-LA after 7 h, 81% *L*-LA conversion was achieved; in the ROP of *rac*-LA, the same monomer conversion was obtained in 3 h. Although this activity is not exceptional, it compares well with those generally reported for the Al complexes. In Figure 2, we compare the activity of (NSO)AlMe_2_ with those of the two Al complexes featuring the prototypical phenoxyimine ligand [43] and the thioether-amide ligands [22]. This last was selected as it displays a different combination of hard and soft donor atoms. Two highly active complexes featuring hydrogen bond donors in the catalytic pocket were also included [30,31].

The analysis of the obtained polymers by means of gel permeation chromatography (GPC) showed that both polymerizations proceed with good control. As a matter of fact, the experimental molecular weights were close to those calculated assuming the growth of one polymer chain for one added alcohol equivalent and the polydispersity indexes (PDI) were monomodal and narrow.

**Figure 3:**
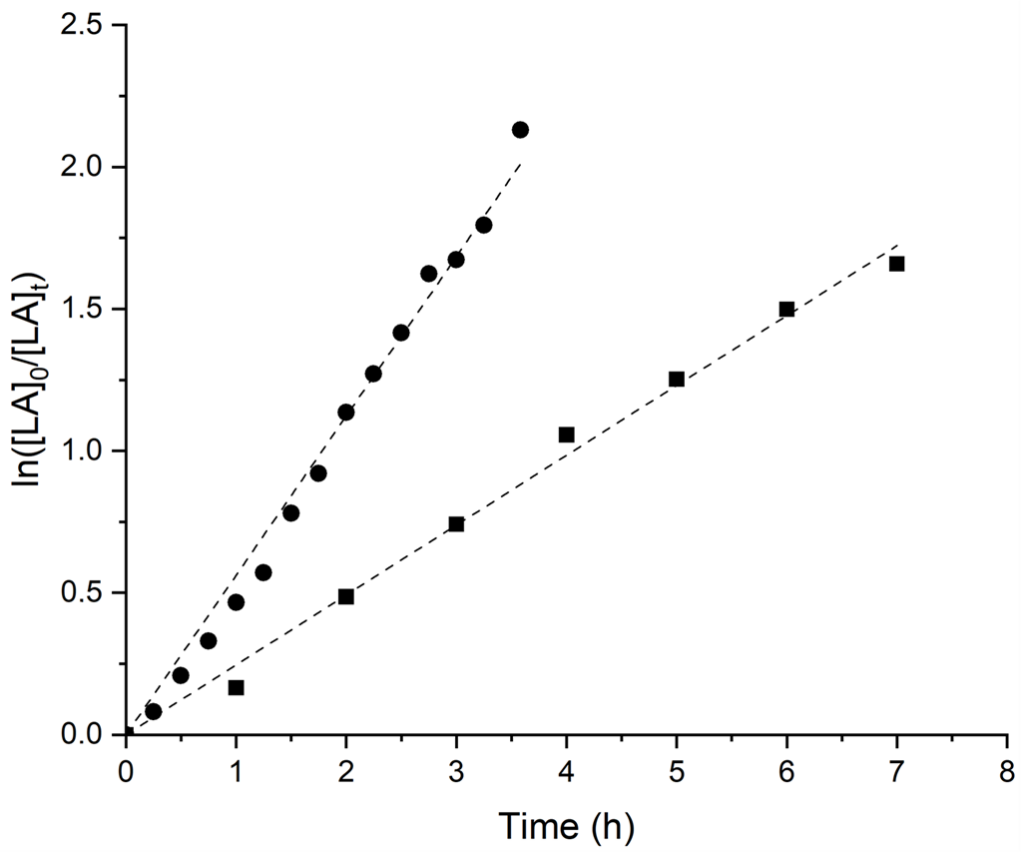
Pseudo-first order kinetic plots for ROP of *L*-LA (*k*_app_ = 0.246 ± 0.004 h^−1^, R^2^ = 0.998, ■) and rac-LA (*k*_app_ = 0.561 ± 0.009 h^−1^, R^2^ = 0.996, ●) by (NSO)AlMe_2_. Conditions: [Al]_0_ = 14 mM, [LA] = 1.4 M, [LA]_0_/[iPrOH]_0_/[cat]_0_ = 100/2/1, toluene as solvent, T = 80 °C.

To have a better insight into the reactivity of (NSO)AlMe_2_ towards the two monomers, the conversions were measured at prescribed polymerization times. Aliquots of reaction mixtures were withdrawn and analyzed by ^1^H NMR spectroscopy; the plots of ln([LA]_0_/[LA]_t_) vs. time are shown in Figure 3. The obtained graphs were linear, indicating that the polymerizations are both of the first order in the monomer concentration. The apparent propagation constants (*k*_app_) were 0.246 ± 0.004 h^−1^ and 0.561 ± 0.009 h^−1^ for *L*-LA and *r*-LA, respectively, confirming that the polymerization of *rac*-LA proceeds at double the rate. The analysis of the microstructure of the produced *rac*-PLA revealed that a heterotactic-enriched PLA (*P*_r_ = 0.59–0.62) was obtained. This suggests a preference for the racemic linkages within the polymer chain and justifies the increased rate of reaction for *rac*-LA versus *L*-LA.

**Figure 4:**
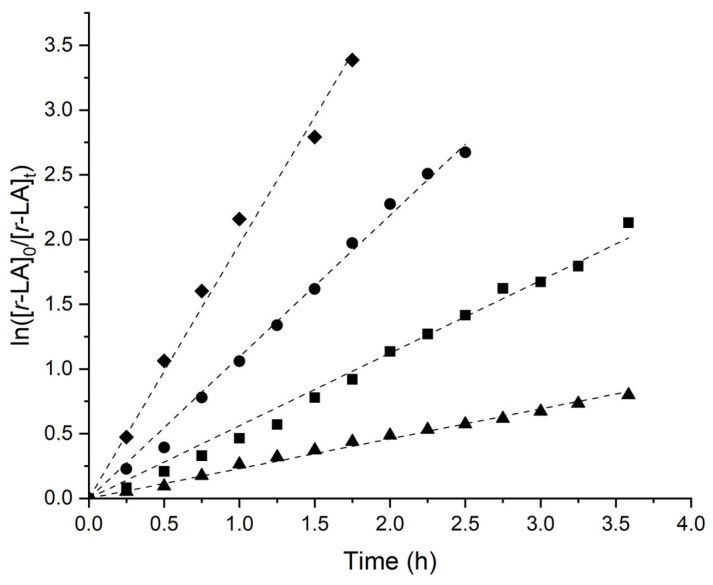
Pseudo-first-order kinetic plots for ROP of rac-LA at different temperatures by (NSO)AlMe_2_. 70 °C (*k*_app_ = 0.230 ± 0.03 h^−1^, R^2^ = 0.998, ▲), 80 °C (*k*_app_ = 0.561 ± 0.004 h^−1^, R^2^ = 0.996, ■), 90 °C (*k*_app_ = 1.09 ± 0.01 h^−1^, R^2^ = 0.998, ●), 100 °C (*k*_app_ = 1.96 ± 0.04 h^−1^, R^2^ = 0.997, ♦). Conditions: [Al]_0_ = 14 mM, [rac-LA] = 1.4 M, [rac-LA]_0_/[iPrOH]_0_/[Al]_0_ = 100/2/1, toluene as solvent.

**Figure 5:**
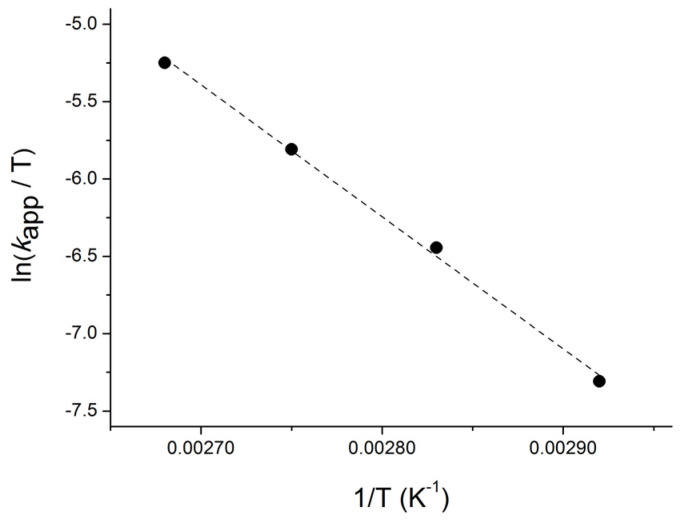
Eyring plot of ln(*k*_app_/T) vs. 1/T. Slope = −8536 ± 293, Intercept = 17.6 ± 0.8. *R*^2^ = 0.99646. ∆*H*^‡^ = 16.9 ± 0.6 kcal/mol kcal mol^−1^, ∆*S*^‡^ = −12 ± 2 cal/mol for ROP of *rac*-LA using (NSO)AlMe_2_/iPrOH.

A deeper investigation of the polymerization of *rac*-LA was carried out. The polymerization temperature was varied between 70 and 100 °C. As expected, an increase in the observed rate resulted in the temperature increasing (70 °C: *k*_app_ = 0.230 h^−1^ < 80 °C: *k*_app_ = 0.561 h^−1^ < 90 °C: *k*_app_ 1.09 h^−1^ < 100 °C: 1.96 h^−1^, Figure 4). At 100 °C, almost quantitative conversion was obtained in 1.5 h. The GPC analysis of the obtained polymers indicated that as the temperature increased, the polymerization control was partially lost: the molecular weight distribution becomes broader and the observed molecular weights tend to deviate from the expected values. The decrease in control is probably related to the advance of transesterification side reactions, for which the rates increase with the temperature, as occurs for the rate of propagation. The polymers isolated from the polymerization carried out at 100 °C were subjected to gel permeation chromatography. The resulted number-average molecular weights increased linearly with the conversion, the PDI values were monomodal, but the values increased during the polymerization, confirming that the propagation step is affected by intra- and intermolecular transesterification side reactions (Figure S9). The results of the variable temperature polymerizations were used to determine the enthalpy (Δ*H*^‡^) and entropy (Δ*S*^‡^) contribution to the activation of the reaction. The thermodynamic parameters, calculated via an Eyring plot, were 16.9 ± 0.6 kcal/mol and −12 ± 2 cal/mol, respectively (Figure 5). The Gibbs free energies of activation (Δ*G*^‡^) were between 21.0 ± 0.1 and 21.4 ± 0.1 kcal/mol for the temperature range tested. These values are comparable to those determined for the industrial standard stannous(II) 2-ethylhexanoate [44]. The values of the enthalpy and entropy of activation are indicative of a more ordered transition state respected by the reactants.

The catalytic performances were investigated in harsher conditions. We first decreased the catalyst concentration (keeping the monomer concentration constant) to increase the [*rac*-LA]/[Al] ratio to 500 and 1000. In these conditions, the catalysts remained active and reached a TOF of 107 or 49 h^−1^ in 4 and 16 h, respectively. Then, we carried out the polymerization under more industrially relevant conditions: in a melt monomer at 190 °C using technical-grade monomers. In these conditions, the complex was able to convert 500 equivalents of *rac*-LA and *L*-LA in 5 min.

To obtain insight into the polymerization mechanism, a low-molecular-weight sample of PLA was prepared. The analysis of the ^1^H NMR spectrum (Figure S12 in the Supporting Information) clearly revealed two quadruplets at 5.04 and 4.33 ppm, attributable to the methine hydrogens of the isopropoxy and lactyl end groups, respectively. The inspection of the MALDI-TOF spectrum confirmed that the PLA are terminated with these end groups. The signals were split by 72 uma, indicating that the propagation step is affected by inter- and intra-transesterification side reactions.

The end group analysis confirms that the reaction starts with the nucleophilic attack of the isopropoxyl group on the carbonyl carbon of the monomer and proceeds through the classical coordination–insertion mechanism, ending with the hydrolysis of the *Al-O* bond between the growing polymer chain and the metal center.

The complex was also tested in the ROP of *ε*-CL and *rac*-*β*-BL in a toluene solution at 80 °C. The representative results are reported in Table 2.

The polymerization of *ε*-CL was faster than the polymerization of *rac*-LA, in accordance with the higher activity of *ε*-CL with respect to LA. Using 1000 monomer equivalents, a high level of conversion was achieved after 5 min of producing polymers with narrow polydispersity and a good agreement between the molecular weights measured by GPC analysis and those calculated theoretically. By decreasing the catalyst concentration in the reaction mixture, high *TOF* values were also reached, but the polymerization control was loosened. A low-molecular-weight sample of polycaprolactone (PCL) was prepared by the conversion of 25 equivalents of the monomer. The analysis of the ^1^H NMR spectrum (see Figure S13 in the Supporting Information) clearly revealed the presence of a signal attributable to isopropoxy-end groups.

**Figure 6:**
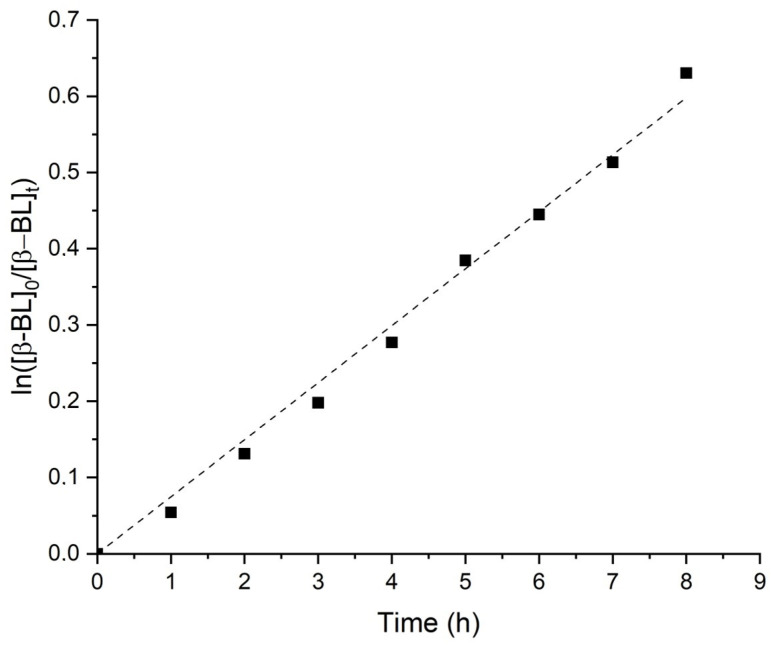
Pseudo-first-order kinetic plots for ROP of *rac*-*β*-BL (*k*_app_ = 0.075 ± 0.001 h^−1^, R^2^ = 0.997). Conditions: [Al]_0_ = 14 mM, [*β*-BL] = 1.4 M, [*β*-BL]_0_/[iPrOH]_0_/[Al]_0_ = 100/2/1, toluene as solvent, T = 80 °C.

The (NSO)AlMe_2_ complex, in combination with iPrOH, was also active in the polymerization of *rac*-*β*-BL. The polymerization proceeded slowly and reached 61% of monomer conversion in 6 h at 100 °C. Despite its high internal strain, *rac*-*β*-BL is relatively inert toward the ROP. A good control of the molecular weight was achieved. A kinetic study for the polymerization of *rac*-*β*-BL was performed as described above for the ROP of *rac*-LA. A plot of ln([*β*-BL]_0_/[*β*-BL]_t_) versus time shows the first-order dependence on the concentration of the monomer, with an apparent propagation rate constant of 0.075 h^−1^ (Figure 6).

### 3.3. Theoretical Investigation

**Figure 7:**
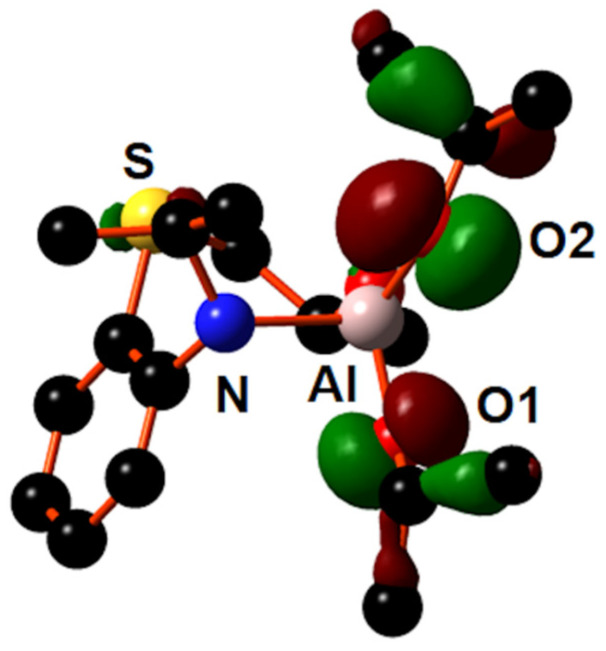
HOMO-1 plot of compound **1_N_**. The hydrogen atoms were hidden for clarity.

DFT calculations were employed to shed light on the mechanism of the polymerization of cyclic esters promoted by (NSO)AlMe_2_. We considered as active species the dialkoxo complex (NSO)Al(OiPr)_2_ that forms from the reaction of the dimethyl aluminum complex with isopropanol. The exchange between the methyl groups, the bond to the Al center and iPrOH is an acid–base reaction and it is relatively fast. Since the activation of the catalyst should not play a dominating role in kinetics, the analysis of the monomers’ activation started from the compound (NSO)Al(OiPr)_2_, namely **1**. To better understand the reactivity of the compound, the electronic structure of **1** was investigated, highlighting the presence near the frontier of a molecular orbital, namely HOMO-1, mainly localized on the oxygen atoms of the alkoxide substituents, as shown in Figure 7.

**Figure 8:**
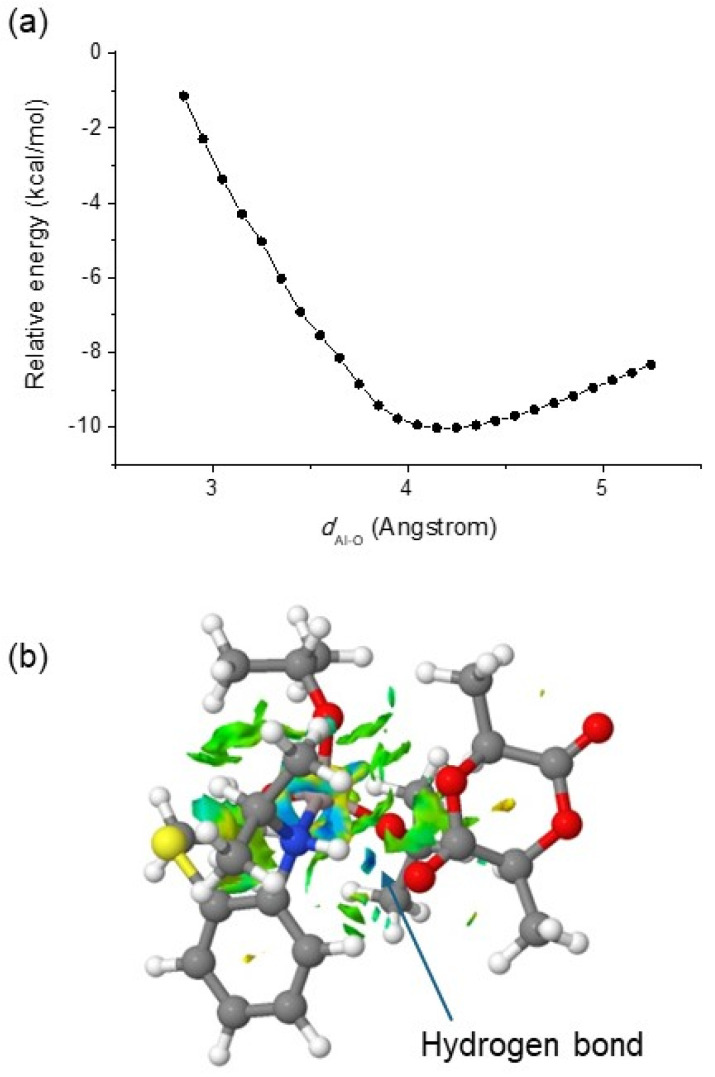
(**a**) Potential energy surface scan for the approach of LA to the metal center of (NSO)Al(OiPr)_2_. (**b**) NCI surface of ***int0*** showing the hydrogen bond between the O atom of lactide and one N-H bond of the ligand.

At the same time, no empty orbital near the frontier of a molecular orbital was found on the Al atom. This hampers the coordination of the monomer on the metal center, as also confirmed by the failure of several attempts to locate a coordination adduct between **1** and LA. In Figure 8a, it is reported that the scans of the energy surface performed upon the approach of LA to the aluminum atom along the direction determined by the Al-O bond, in trans to the oxygen atom. The graph shows a relative minimum when the exocyclic oxygen atom of LA is placed at about 4.2 Å; the relaxed optimization of this structure converged to a stable adduct, namely ***int0***, in which the exocyclic oxygen atom is at 4.19 Å from the metal center and, at the same time, at 2.07 Å from the hydrogen atom of the NH group.

The presence of the short H···O distances suggests that LA and **1** are bonded through a hydrogen bond. Non-covalent interaction (NCI) surfaces [39,40] were created and confirmed the presence of a hydrogen bond between an O atom of lactide and the N-H bond of the ligand (Figure 8b). The binding energy (Δ*H_bind_*), calculated as the difference between the enthalpy of the **1**/LA adduct and the sum of the enthalpies of **1** and LA, is −8.3 kcal/mol.

**Figure 9:**
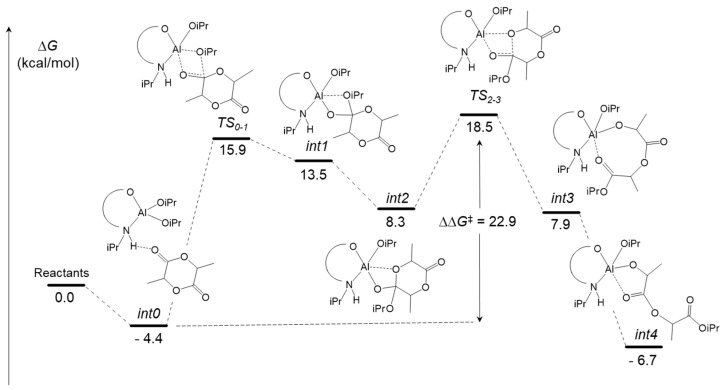
Potential energy surface (PES) corresponding to first insertion of *L*-LA.

**Figure 10:**
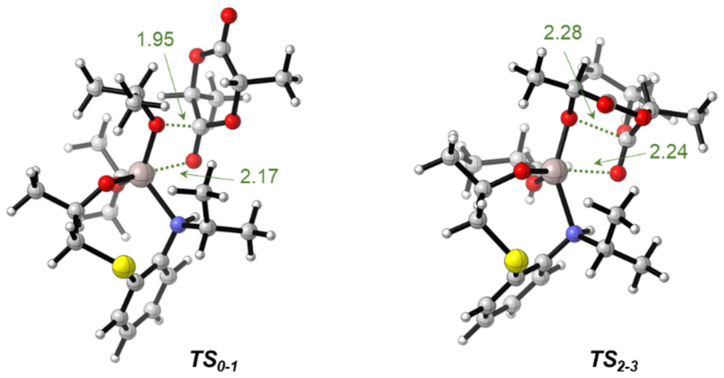
Minimum energy structures for the transition states ***TS_0–1_*** and ***TS_2–3_***. The distances are given in Angstrom.

Taking ***int0*** as the starting point, the reaction proceeds to follow a two-step mechanism (Figure 9). In the first step, the system evolves through a Transition State *TS_0–1_*, as shown in Figure 10, with a free energy barrier of 20.3 kcal/mol. From a structural viewpoint, the exocyclic oxygen atom of LA is located between the metal center (*d*_Al-O_ = 2.17 Å) and the hydrogen of the NH group (*d*_O-H_ = 2.05 Å). The hydrogen bond promotes the monomer activation by decreasing the electron density on the carbon atom of the carbonyl. After ***TS_0–1_***, the orthoalkoxide intermediate ***int1*** is obtained. The anion formed by this nucleophilic attack replaces the isopropoxy group in the coordination sphere of the Al atom. To let the intracyclic oxygen atom of the LA come closer to the metal center, ***int1*** rearranges in the more stable intermediate ***int2***. From this, the ring opening takes place via ***TS_2–3_*** (Figure 9) with a free-energy barrier of 22.9 kcal/mol and affords the intermediate ***int3*** at 7.9 kcal/mol. Finally, the isomerization of the growing chain leads to the stable heterocyclic Al lactate product (***int4***), making the ring-opening reaction of the LA thermodynamically favored. The opening of the ring (***TS_2–3_***) represents the rate-determining step of the whole process, the value of the activation barrier compares well with the Gibbs free energies of activation, experimentally determined through the Eyring plot. Its value justifies the necessity of carrying out the polymerization reaction at a high temperature.

A parameter that has been recently used to rationalize the activity of Al catalysts is the framework distortion energy (FDE) [45]. This represents the energy cost that the complex must pay to distort its ligand geometry and to adopt the structure of the rate-determining transition state. In the case of our complex, the FDE was estimated to be 13.2 kcal/mol, quite a large value compared with the FDE values reported for the active Al complexes investigated for cyclic ester ROP [45]. As an example, the FDE calculated for the highly active Al(III) complexes bearing the catechol–amine ligands was 5.8 kcal/mol [30]. The close inspection of the geometries of the complex and ***TS_2–3_*** revealed that the aluminum atom changes its coordination geometry from a tetrahedral coordination geometry in the starting complex to a distorted trigonal bipyramidal coordination geometry in ***TS_2–3_***. The increase in the coordination number is probably at the origin of the high energy cost that the complex has to pay in reaching the rate-determining transition state.

**Figure 11:**
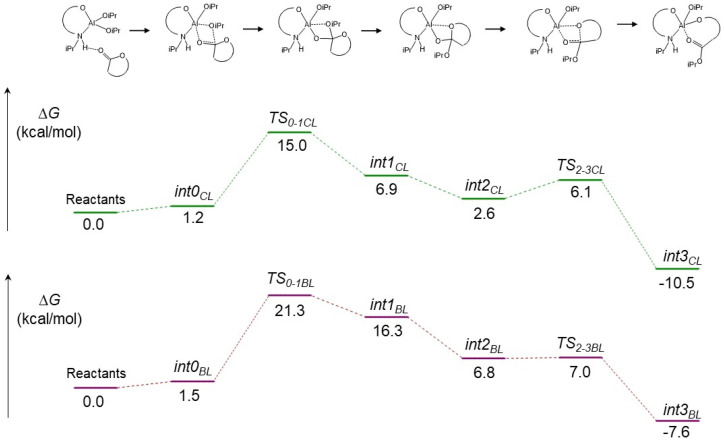
Potential energy surface (PES) corresponding to the first insertion of *ε*-CL (**top**) and *β*-BL (**down**).

The free energy reaction pathways for the ring-opening polymerization of *ε*-CL and *β*-BL were also computationally addressed. The corresponding reaction profiles are reported in Figure 11. Similar to the previous case, all the efforts to localize a starting intermediate featuring the direct coordination of *ε*-CL or *β*-BL to the Al atom failed. In both cases, a starting adduct was located (***int0_CL_*** and ***int0_BL_***) in which the monomer is docked on the ligand through a hydrogen bond between the oxygen atom of the carbonyl group and the NH functional group. On the free-energy surface, the formation of these structures is slightly disfavored, probably in view of the entropy decrease due to their formation. The binding energies (Δ*H_bind_*) for ***int0_CL_*** and ***int0_BL_***, calculated as the difference between the enthalpy of the adduct and the sum of the enthalpies of **1** and the monomer, were −5.7 kcal/mol and −6.4 kcal/mol, respectively.

Starting from ***int0_CL_*** (or ***int0_BL_***), the reaction proceeds, overcrossing two transition states. In the first step, the isopropoxide oxygen nucleophilically attacks the carbonyl carbon of the monomer (***TS_0–1CL_*** or ***TS_0–1BL_***), leading to the orthoalkoxide intermediate ***int1_CL_*** (or ***int1_BL_***). This rearranges into the second intermediate ***int2_CL_*** (or ***int2_BL_***) that subsequently overcomes the transition state corresponding to the ring-opening of the monomer (***TS_2–3CL_*** or ***TS_2–3BL_***). The formation of ***int3_CL_*** (or ***int32_BL_***) is exergonic by −10.5 kcal/mol (or −7.6 kcal/mol), making the growth of the polymer chain thermodynamically feasible.

Differently from the case of the LA polymerization, the rate-determining step is the nucleophilic attacks to the carbonyl carbon of the monomer in both cases. The Δ*G*^‡^ for the *ε*-CL insertion is 15.0 kcal/mol, whereas the Δ*G*^‡^ for the *β*-BL insertion is 21.3 kcal/mol. These values are coherent with the relative reactivity displayed by these monomers in the ROP promoted by (NSO)AlMe_2_/iPrOH.

## 4. Conclusions

In this work a new aluminum complex, (NSO)AlMe_2_, featuring an aminophenyl–thio-propoxide ligand has been synthesized and characterized by NMR spectroscopy. The ligand coordinates to the metal center through the anionic oxygen atom and neutral nitrogen atom, affording a distorted tetrahedral complex. The amine function is stable and does not react with the Al–carbon bond, even at high temperatures.

In the presence of isopropanol, the complex (NSO)AlMe_2_ was able to promote the ring-opening polymerization of *L*-, *rac*-LA, *ε*-CL and *β*-BL. The polymerization of the *rac*-LA was faster than that of *L*-LA: high monomer conversion was reached in 3 h of polymerization, affording a heterotactic-enriched PLA. The polymerization process was controlled and allowed the synthesis of polymers with prescribed molecular weights and narrow polydispersities. Kinetic studies showed that the polymerization follows a first-order kinetic law with respect to the monomer concentration; the enthalpy (Δ*H*^‡^) and entropy (Δ*S*^‡^) of activation were also determined through the Eyring plot. The catalytic system retained its activity at a very low concentration and in the melt using technical-grade monomers. A relatively high level of activity was observed in the ring-opening polymerization of *ε*-CL. DFT calculations revealed the pivotal role played by the coordinated ligand: the NH function, acting as a hydrogen bond donor, docks the monomer in the proximity of metal center and activates the monomer toward the nucleophilic attack of the growing polymer chain.

The results reported in this work demonstrate that aluminum complexes featuring a hydrogen bond donor on the coordination sphere represent good candidates to develop new catalytic systems for the ring-polymerization of cyclic esters. We think that our experimental and theoretical studies can contribute to deepening the understanding of the relationships between the characteristics of the coordinated ligand at the metal center and the reactivity of the complex in the polymerization of cyclic esters.

## Figures and Tables

**Table 1:** Ring-opening polymerization of *rac*- and *L*-LA promoted by (NSO)AlMe_2_/iPrOH.

Run ^(a)^	Mon.	[Mon]/[Al]	T [°C]	t [h]	Conver. [%] ^(b)^	TOF [h^−1^] ^(c)^	Mn_(th)_ [kg/mol] ^(e)^	Mn_(exp)_ [kg/mol] ^(f)^	PDI ^(f)^
1	*L*-LA	100	80	7	81	12	5.8	5.6	1.08
2	*rac*-LA	100	80	3	81	27	5.8	5.8	1.22
3	*rac*-LA	100	90	1.5	80	53	5.8	4.3	1.46
4	*rac*-LA	100	100	1.5	94	63	6.8	4.3	1.53
5	*rac*-LA	500	80	4	86	107	30.9	28.7	1.36
6	*rac*-LA	1000	80	16	78	49	56.7	52.4	1.41
7 ^(d)^	*rac*-LA	1000	190	0.08	56	563	40.3	38.9	1.07
8 ^(d)^	*L*-LA	1000	190	0.08	52	522	37.5	16.2	1.30

^(a)^ All reactions were carried out in 2.0 mL of toluene as solvent, [Al]_0_ = 14 mM, [LA] = 1.4 M, [LA]_0_/[iPrOH]_0_/[cat]_0_ = 100/2/1; ^(b)^ Molecular conversion determined by ^1^H NMR spectroscopy (CDCl_3_, 298 K); ^(c)^ TOF = mol_Mon_ × conversion/(mol_Al_ × h); ^(d)^ Polymerization without solvent; ^(e)^ Calculated molecular weight using M_n(th)_ = (144.13 × ([LA]_0_/[iPrOH]_0_) × conversion)/1000; ^(f)^ Experimental molecular weight M_n(exp)_ and polydispersity index (PDI) determined by GPC in THF using polystyrene standards and corrected using factor 0.58.

**Table 2:** Ring-opening polymerization of *ε*-CL and *rac*-*β*-BL promoted by (NSO)AlMe_2_/iPrOH.

Run ^(a)^	Mon.	[Mon]/[Al]	t [min]	Conv. [%] ^(b)^	TOF [h^−1^] ^(c)^	Mn_(th)_ [kg/mol] ^(e)^	Mn_(expt)_ [kg/mol^−1^]	PDI ^(f)^
1	*ε*-CL	1000	5	77	9254	43.9	42.6	1.10
2	*ε*-CL	2000	10	78	9389	89.0	66.3	1.13
3	*ε*-CL	4000	10	53	12766	121.0	27.6	1.72
4	*ε*-CL	4000	40	81	4878	184.9	33.4	2.02
5	*β*-BL	100	240	24	6	1.0	n.d.	n.d.
6 ^(d)^	*β*-BL	100	240	61	15	2.6	3.0	1.21

^(a)^ All reactions were carried out in 2.0 mL of toluene as solvent, [Al]_0_ = 14 mM, [Mon] = 1.4 M, [Mon]_0_/[iPrOH]_0_/[cat]_0_ = 100/2/1, T = 80 °C. ^(b)^ Molecular conversion determined by ^1^H NMR spectroscopy (CDCl_3_, 298 K). ^(c)^ TOF = mol_Mon_ × conversion/(mol_Al_ × h). ^(d)^ T = 100 °C. ^(e)^ Calculated molecular weight using M_n(th)_ = (PM_Mon_ × ([Mon]_0_/[iPrOH]_0_) × conversion)/1000. ^(f)^ Experimental molecular weight M_n(expt)_ and polydispersity index (PDI) determined by GPC in THF using polystyrene standards and corrected using the factor 0.56 for PCL and 0.54 for PHB.

## Data Availability

The data that support the findings of this study are available from corresponding author, upon reasonable request.

## Supplementary Materials

The following supporting information can be downloaded at: https://www.mdpi.com/article/10.3390/polym16213047/s1, Figures giving NMR spectra of NSO-H and (NSO)AlMe_2_, ^1^H NMR spectra of the obtained polymers, cartesian coordinates and free energies of all the structures optimized in the computational analysis.
