# Supplementary material for: Hydrogen Bond Donors in the Catalytic Pocket: The Case of the Ring-Opening Polymerization of Cyclic Esters Catalyzed by an Amino-Propoxide Aluminum Complex
Source: Polymers (Basel). 2024 Oct 30;16(21):3047. doi: 10.3390/polym16213047 (PMC11548642; doi:10.3390/polym16213047)
Supplement: Supplementary file 1 [file polymers-16-03047-s001.zip › polymers-3249026-supplementary.pdf]

Supporting Information

Hydrogen Bond Donors in the Catalytic Pocket: The Case of the Ring-Opening Polymerization of Cyclic Esters Catalyzed by an Amino-Propoxide Aluminum Complex

Salvatore Impemba [1], Antonella Viceconte [1], Irene Tozio [1], Shoaib Anwar [1], Gabriele Manca [2,*] and Stefano Milione [1,*]

[1] Department of Chemistry and Biology, University of Salerno, Via Giovanni Piano II, 84084 Fisciano, Salerno, Italy; simpemba@unisa.it (S.I.); antonellaviceconte97@gmail.com (A.V.); just_irene@live.it (I.T.); sanwar@unisa.it (S.A.)

[2] CNR-ICCOM, Consiglio Nazionale delle Ricerche, Via Madonna del Piano, 10, 50019 Sesto Fiorentino, Firenze, Italy

* Correspondence: gmanca@iccom.cnr.it (G.M.); smilione@unisa.it (S.M.)

Contents

	pag.
**Experimental section**	*S3*
Figure S1: ^{1}H NMR of NSO-H.	*S4*
Figure S2: ^{13}C NMR of NSO-H.	*S4*
Figure S3: HSQC NMR spectrum of NSO-H.	*S5*
Figure S4: Maldi-MS spectrum of NSO-H.	*S5*
Figure S5: ^{1}H NMR of (NSO)$AlMe_2$.	*S6*
Figure S6: ^{13}C NMR of (NSO)$AlMe_2$.	*S6*
Figure S7: COSY NMR spectrum of (NSO)$AlMe_2$.	*S7*
Figure S8: HSQC NMR spectrum of (NSO)$AlMe_2$.	*S8*
Figure S9. Plot of number-averaged molecular weight Mn *vs.* monomer conversion using (NSO)$AlMe_2$/iPrOH as catalyst.	*S9*
Figure S10: ^{1}H NMR of PLA obtained using (NSO)$AlMe_2$/iPrOH as catalyst.	*S9*
Figure S11: ^{13}C NMR of PLA obtained using (NSO)$AlMe_2$/iPrOH as catalyst	*S10*
Figure S12: ^{1}H NMR of oligomers of PLA obtained using (NSO)$AlMe_2$/iPrOH	*S10*
Figure S13: ^{1}H NMR of oligomers of PCL obtained using (NSO)$AlMe_2$/iPrOH .	*S11*
Figure S14: ^{1}H NMR of PHB obtained using (NSO)$AlMe_2$/iPrOH as catalyst.	*S11*
Figure S15: ^{13}C NMR of PHB obtained using *(*NSO*)*$AlMe_2$/iPrOH as catalyst.	*S12*
Figure S16: SEC of PLA obtained using (NSO)$AlMe_2$/iPrOH as catalyst.	*S12*
Figure S17. Methine region of the homonuclear decoupled ^{1}H[^{1}H] NMR spectrum for *rac*-PLA obtained by (NSO)$AlMe_2$/iPrOH as catalyst	*S13*
Cartesian coordinates and free energies of all the structures optimized in the computational analysis	*S14*

Experimental section

Materials and methods. All preparations and subsequent manipulation of air- and/or water-sensitive compounds were carried out under a dry nitrogen atmosphere using a Braun Labmaster drybox or standard Schlenk line techniques. Glassware and vials used in the polymerization were dried in an oven at 120 °C overnight and exposed three times to vacuum-nitrogen cycles. All solvents and reagent were dried and purified before use. Toluene (Sigma-Aldrich, 99.5%) and hexane (Sigma-Aldrich, 99%) were heated to reflux for 48 h over sodium or sodium ketyls and distilled before use for moisture- and oxygen-sensitive reactions. All other solvents were used as received (TCI or Sigma-Aldrich) or distilled under reduced pressure over calcium hydride. Ligands used for the synthesis of complexes were dried in vacuum with P_2O_5. *Rac*-β-butyrolactone (βBL), ε-caprolactone (εCL) and isopropyl alcohol (iPrOH) were dried over CaH_2 one night and freshly distilled under reduced pressure. *L*- and *rac*-lactide was purified by recrystallization from toluene twice and subsequently dried over P_2O_5 under dynamic vacuum and finally stored in the glove box. Deuterated solvents were purchased from Sigma-Aldrich dried over activated 4Å molecular sieves prior to use.

Instruments and Measurements. The NMR spectra were collected by using Bruker Avance spectrometers (400.13 MHz for ^{1}H and 100.62 MHz for ^{13}C). Chemical shifts (δ) are listed as parts per million and coupling constants (*J*) in Hertz. ^{1}H NMR spectra are referenced using the residual solvent peak at δ 7.16 for C_6H_6, δ 7.27 for $CDCl_3$, δ 5.32 for CD_2Cl_2 and δ 6.00 for $C_2D_2Cl_4$. ^{13}C NMR spectra are referenced using the residual solvent peak at δ at 128.39 for C_6H_6, δ 77.23 for $CDCl_3$, δ 53.84 for CD_2Cl_2 and δ 73.78 for $C_2D_2Cl_4$. *High-Resolution Matrix Assisted Laser Desorption Ionization Analysis.* In a general procedure of MALDI-TOF MS sample preparation a 1.0 mg of substance was dissolved in 1.0 mL of CH_2Cl_2. 4 µL of this solution was added in 45 µL of solution of dihydroxybenzoic acid 40 mM in CH_2Cl_2 as matrix agent. Molecular weights (M_n and M_w) and molecular weight distributions (PDI = M_w/M_n) of polymer samples were measured by using gel permeation chromatography (GPC) at 30 °C with THF as the solvent, a flow rate of the eluent of 1 mL/min, and narrow polystyrene standards as the references. The measurements were performed by using a Waters 1525 binary system equipped with a Waters 2414 refractive index detector and four Styragel columns (range 1000–1000000). DSC thermogram was recorded by using a DSC Q2000 (TA Instruments) under a N_2 flow with a heating and cooling rate of 10 °C/min.

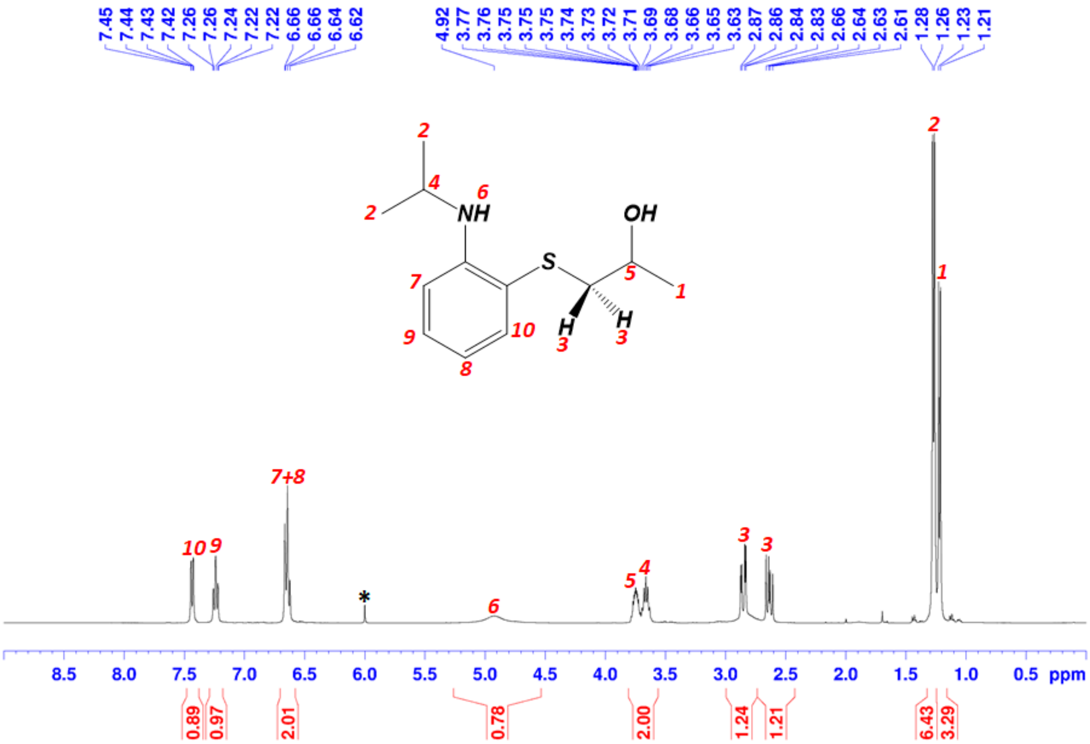

Figure S1: ^{1}H NMR of NSO-H (400.13 MHz, $^{*}C_2D_2Cl_4$, 25 °C).

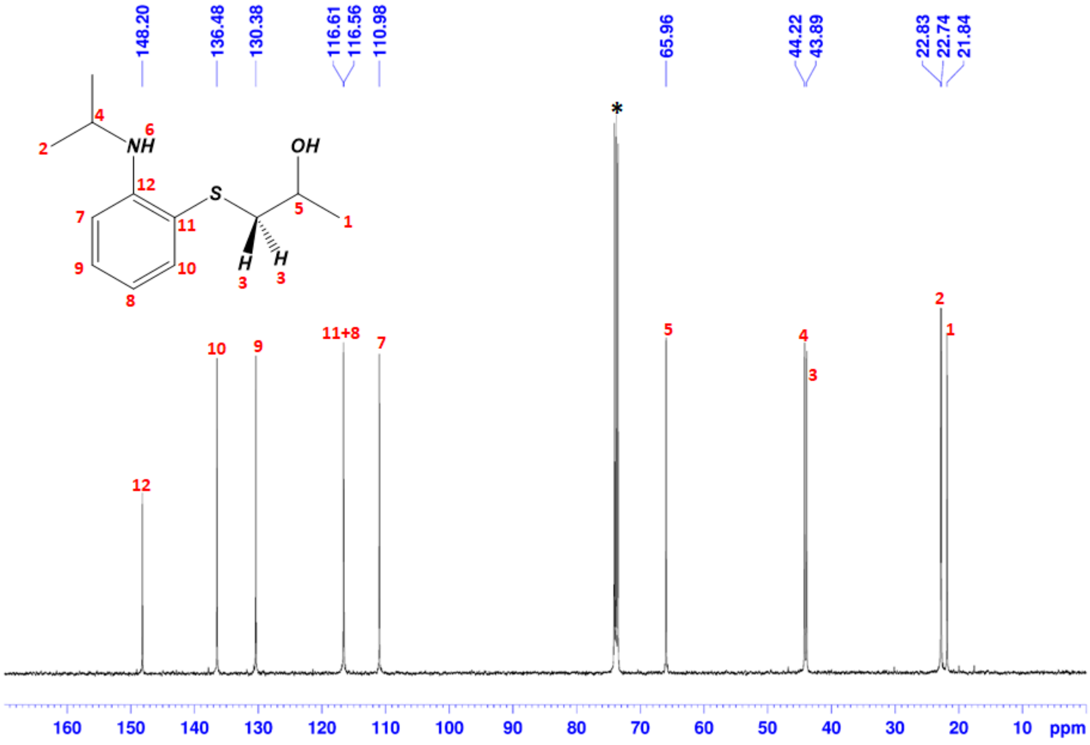

Figure S2: ^{13}C NMR of NSO-H (100.62 MHz, $^{*}C_2D_2Cl_4$, 25 °C).

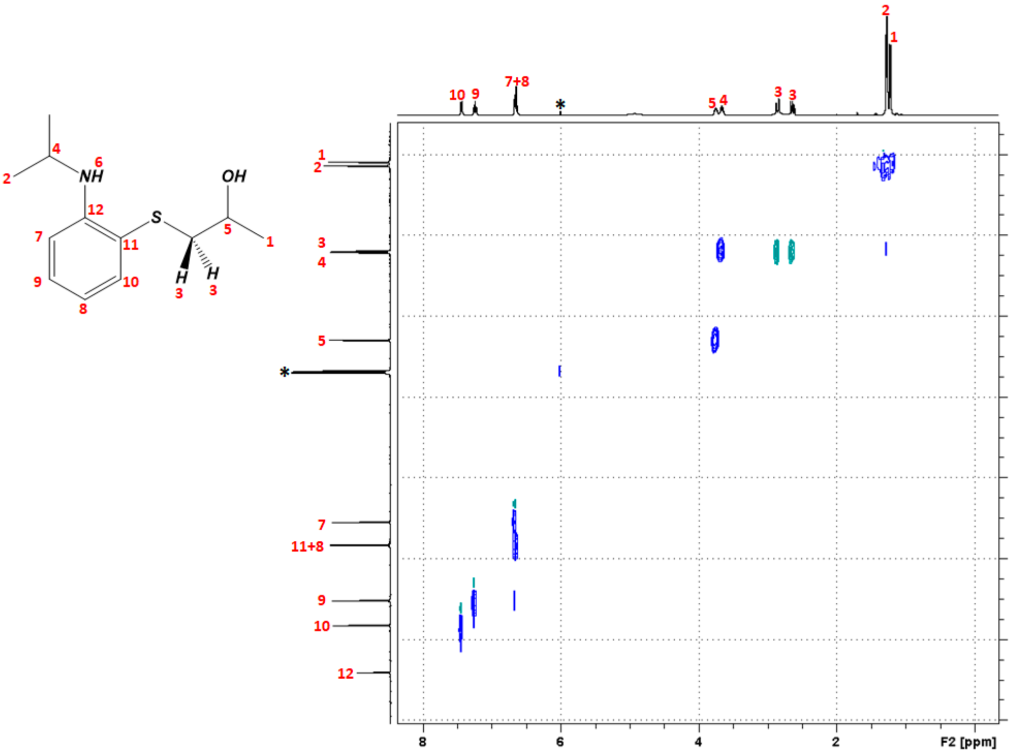

Figure S3: HSQC of NSO-H ($^{*}C_2D_2Cl_4$, 25 °C).

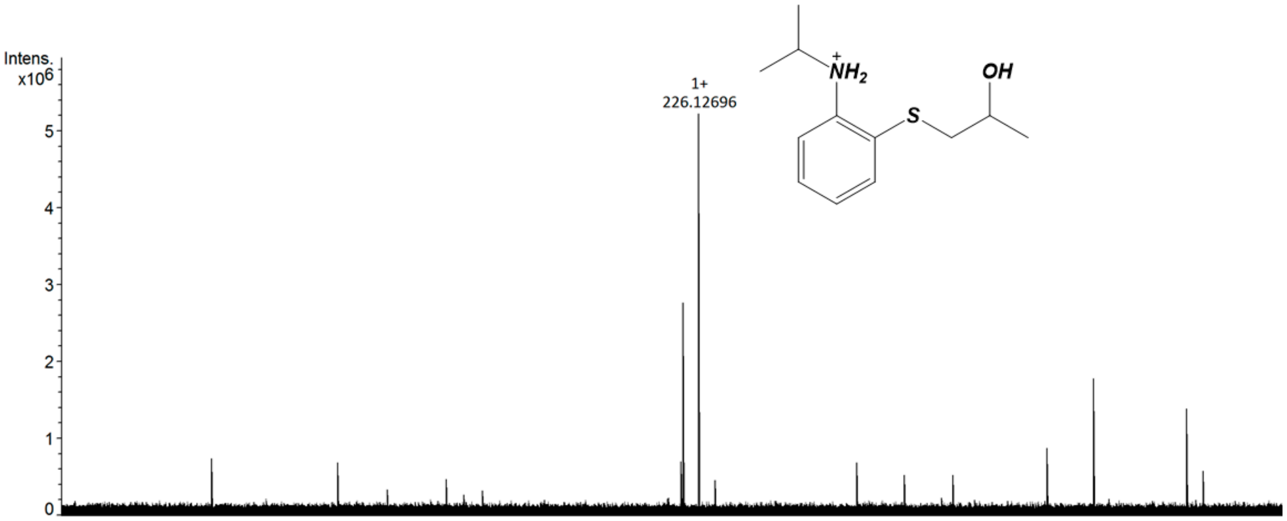

Figure S4: Maldi-MS spectrum of NSO-H

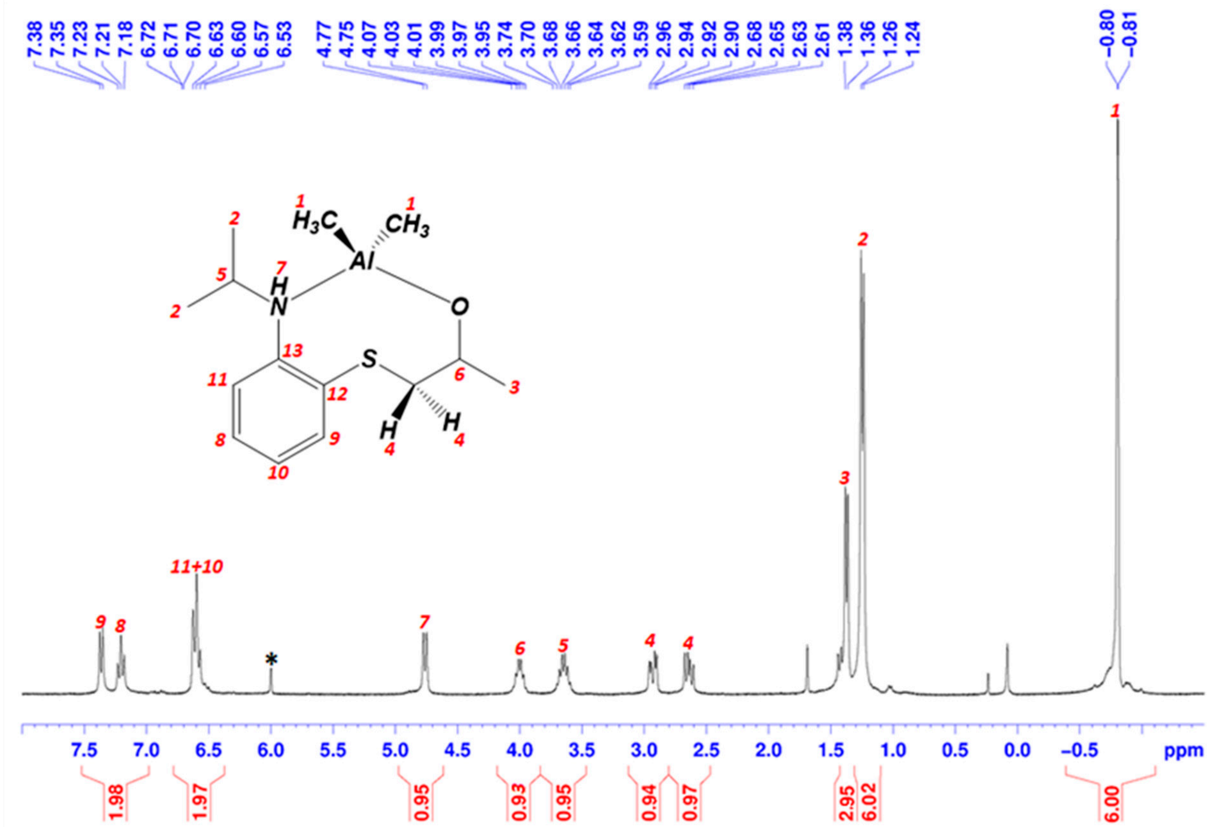

Figure S5: ^{1}H NMR of (NSO)$AlMe_2$ (400.13 MHz, $^{*}C_2D_2Cl_4$, 25 °C).

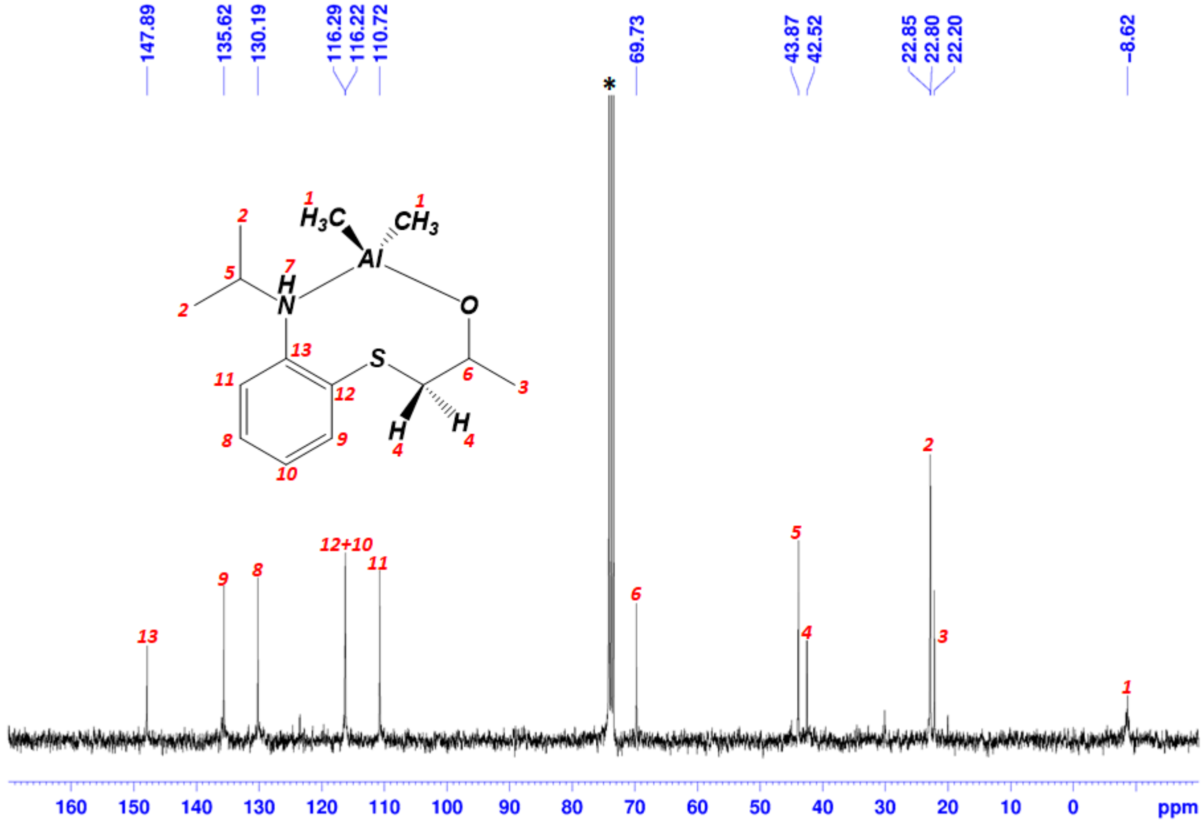

Figure S6: ^{13}C NMR of (NSO)$AlMe_2$ (100.62 MHz, $^{*}C_2D_2Cl_4$, 25 °C).

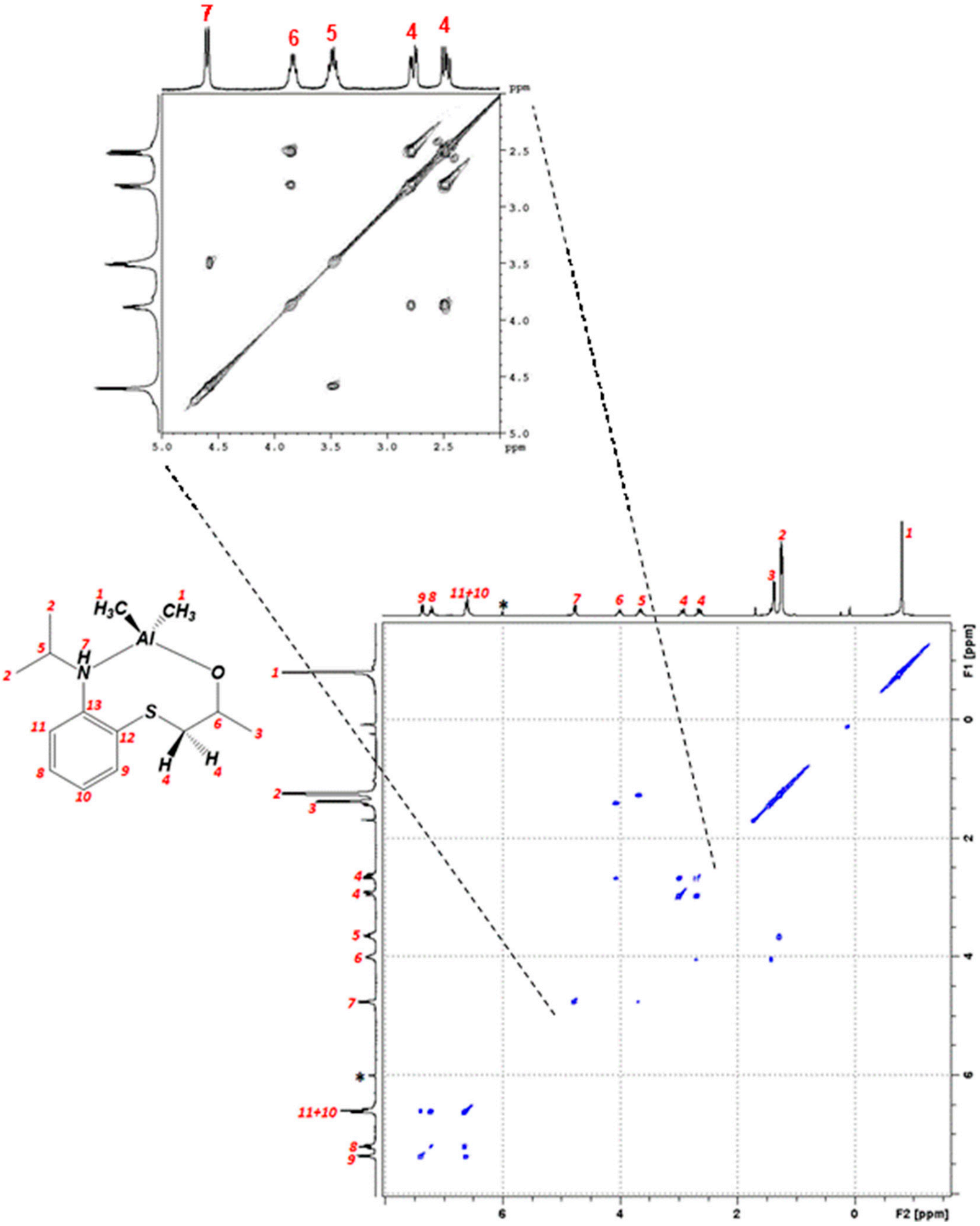

Figure S7: COSY NMR of (NSO)AlMe$_2$ (400.13 MHz, *$C_2D_2Cl_4$, 25 °C).

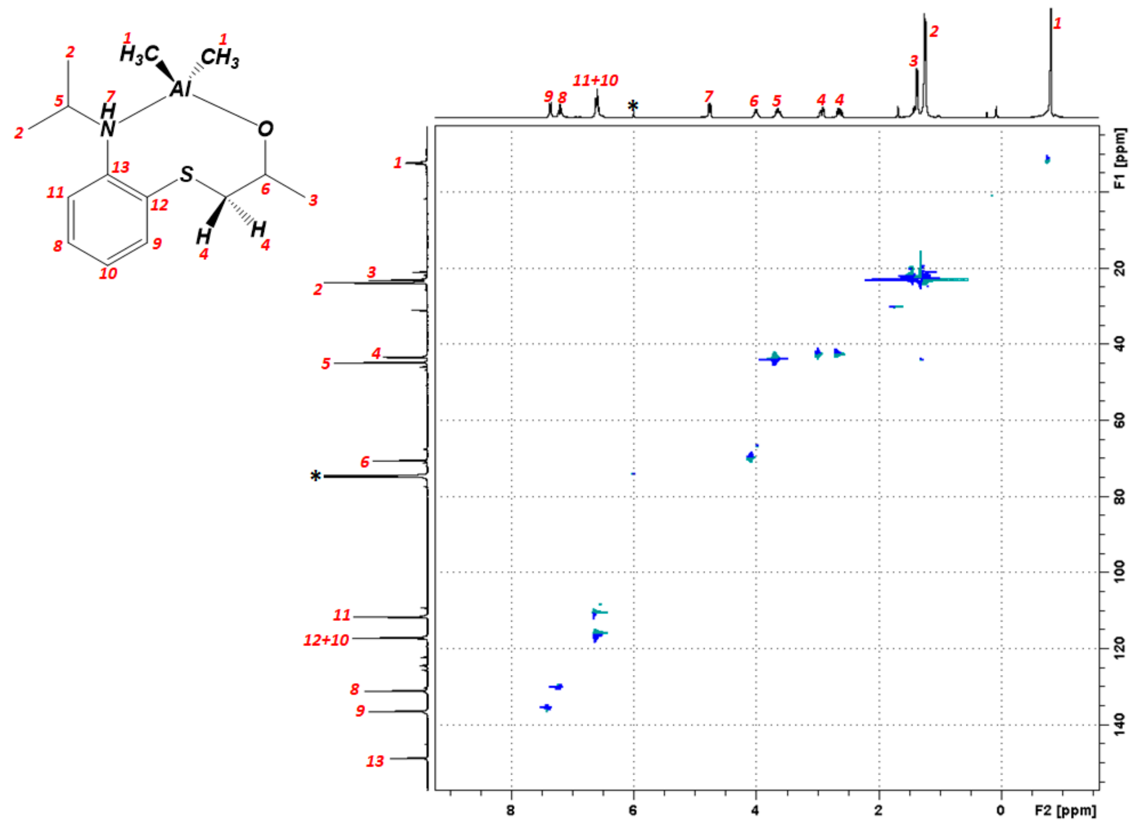

Figure S8: HSQC NMR of (NSO)AlMe$_2$ ($^*C_2D_2Cl_4$, 25 °C).

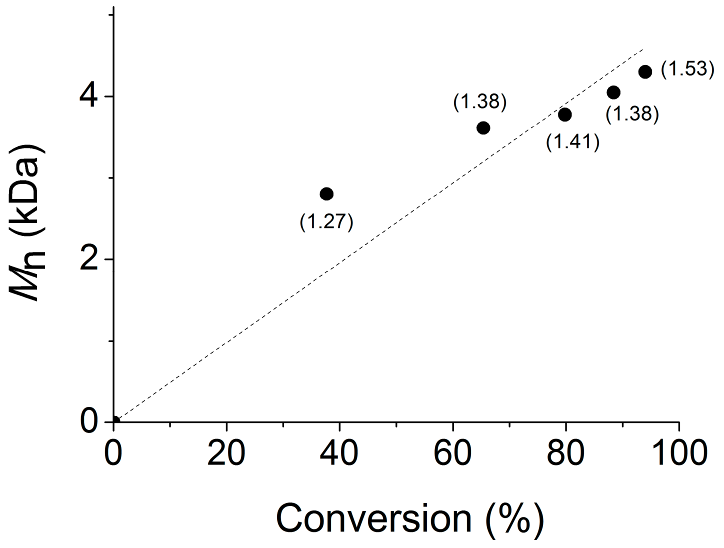

Figure S9. Plot of number-averaged molecular weight *M*n *vs*. monomer conversion using (NSO)AlMe2/iPrOH as catalyst. Conditions: $[Al]_0$ = 14 mM, [LA] = 1.4 M, $[LA]_0/[iPrOH]_0/[cat]_0$ = 100/2/1, toluene as solvent, T = 100 °C.

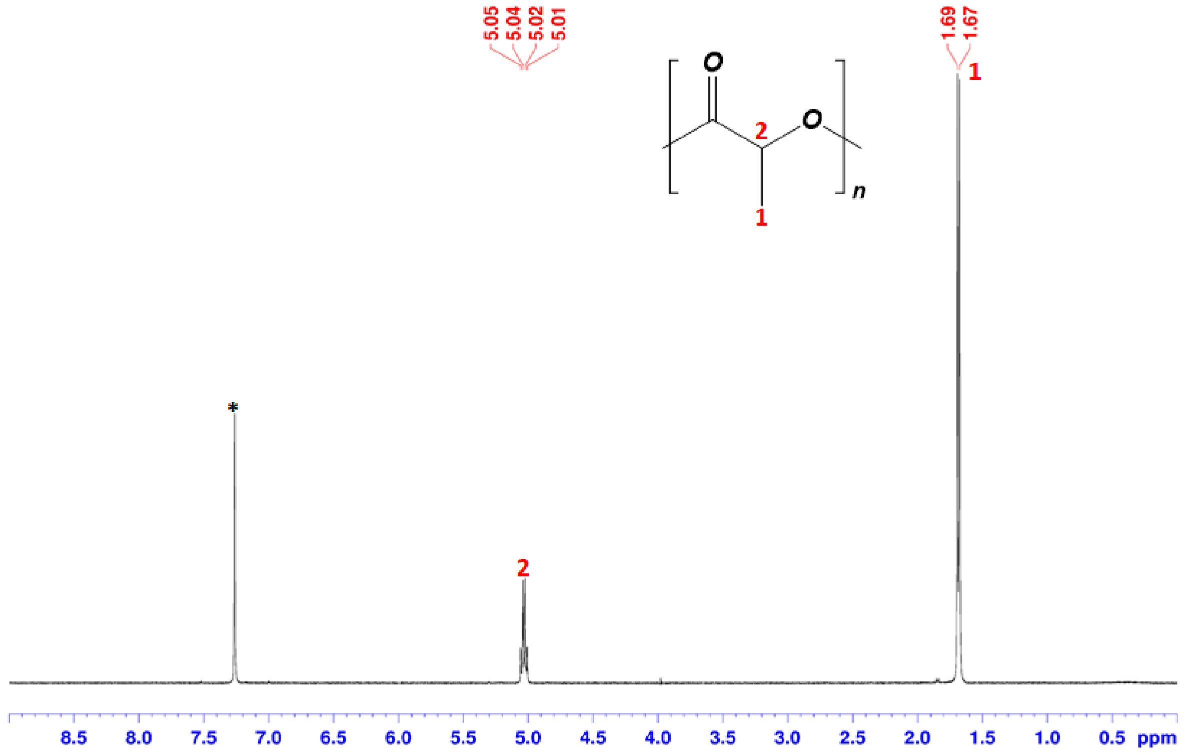

Figure S10: ^{1}H NMR of PLA obtained using (NSO)AlMe2/iPrOH as catalyst (**Run 1, Table 1**), (400.13 MHz, *CDCl3, 25°C).

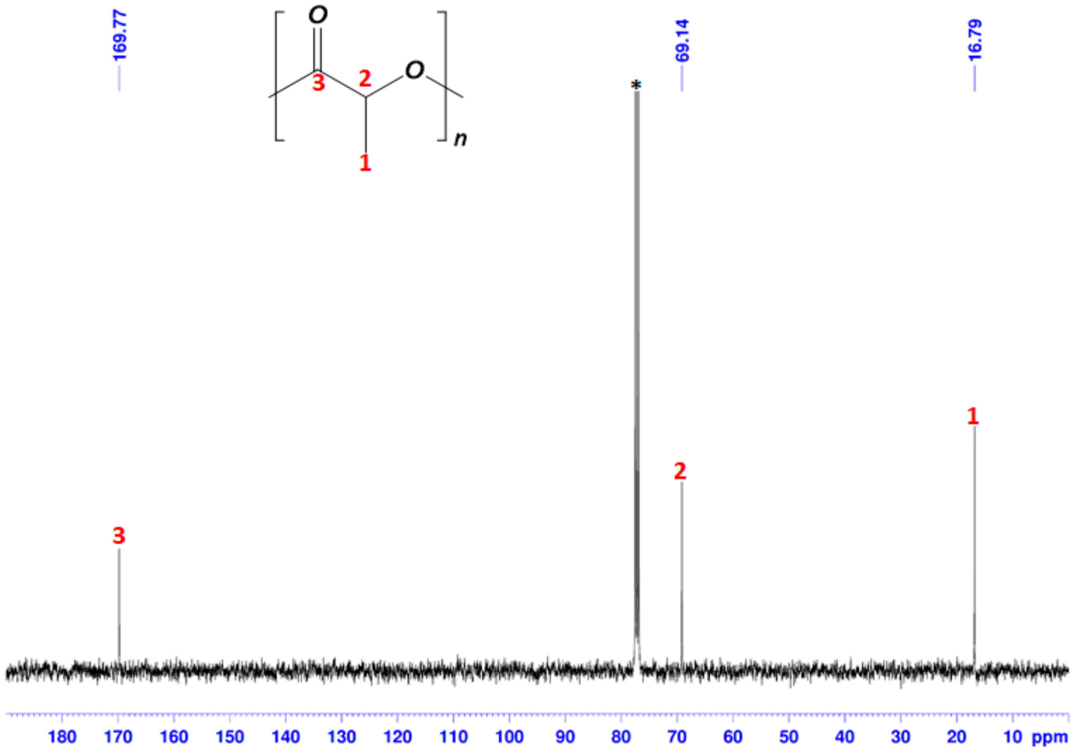

Figure S11: ^{13}C NMR of PLA obtained using (NSO)AlMe$_2$/iPrOH as catalyst (**Run 1, Table 1**), (100.62 MHz, *CDCl$_3$, 25°C).

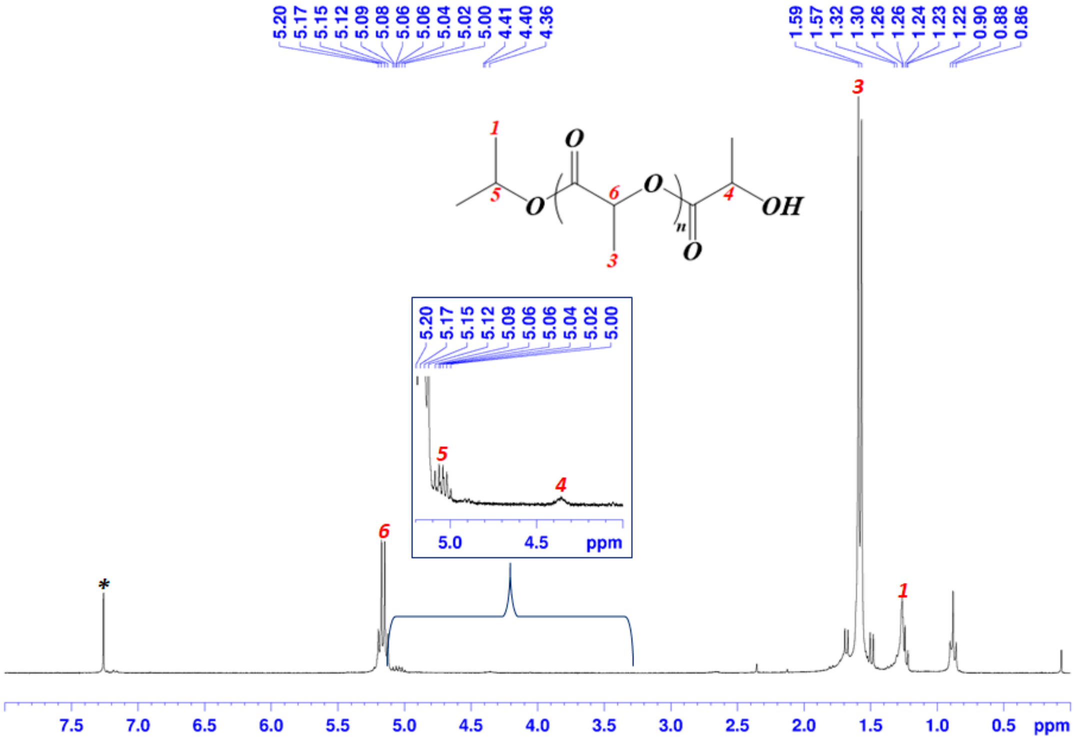

Figure S12: ^{1}H NMR of oligomers of PLA obtained using (NSO)AlMe$_2$/iPrOH as catalyst (400.13 MHz, *CDCl$_3$, 25°C).

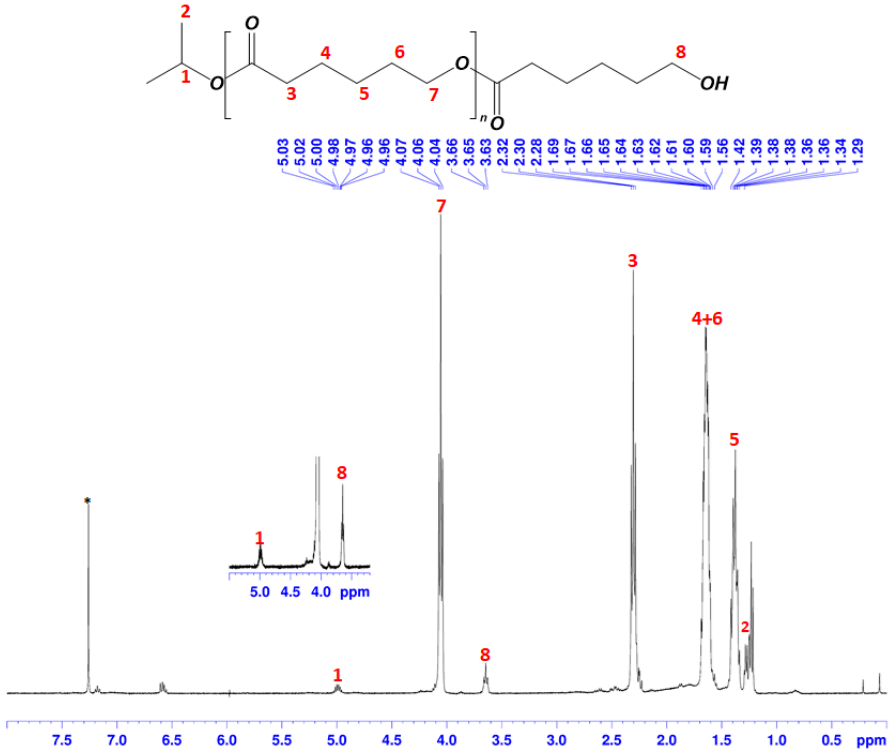

Figure S13: ^{1}H NMR of oligomers of PCL obtained using (NSO)$AlMe_2$/iPrOH as catalyst (400.13 MHz, *$CDCl_3$, 25°C).

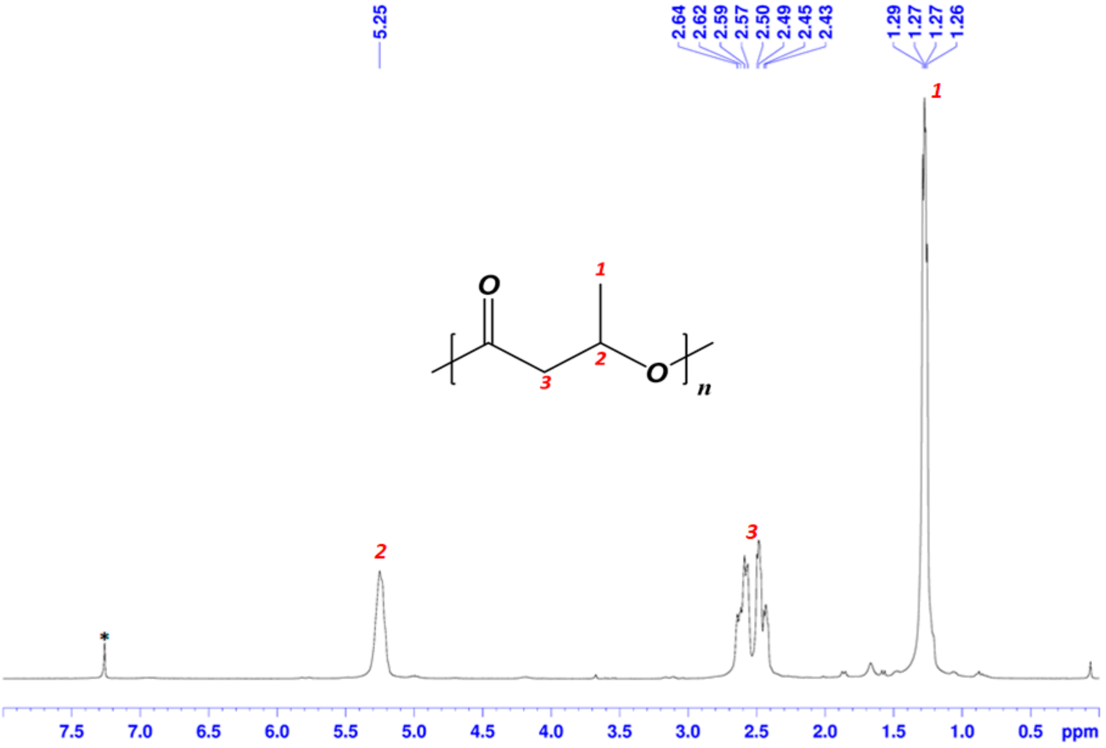

Figure S14: ^{1}H NMR of PHB obtained using (NSO)$AlMe_2$/iPrOH as catalyst (**Run 5, Table 2**), (400.13 MHz, *$CDCl_3$, 25°C).

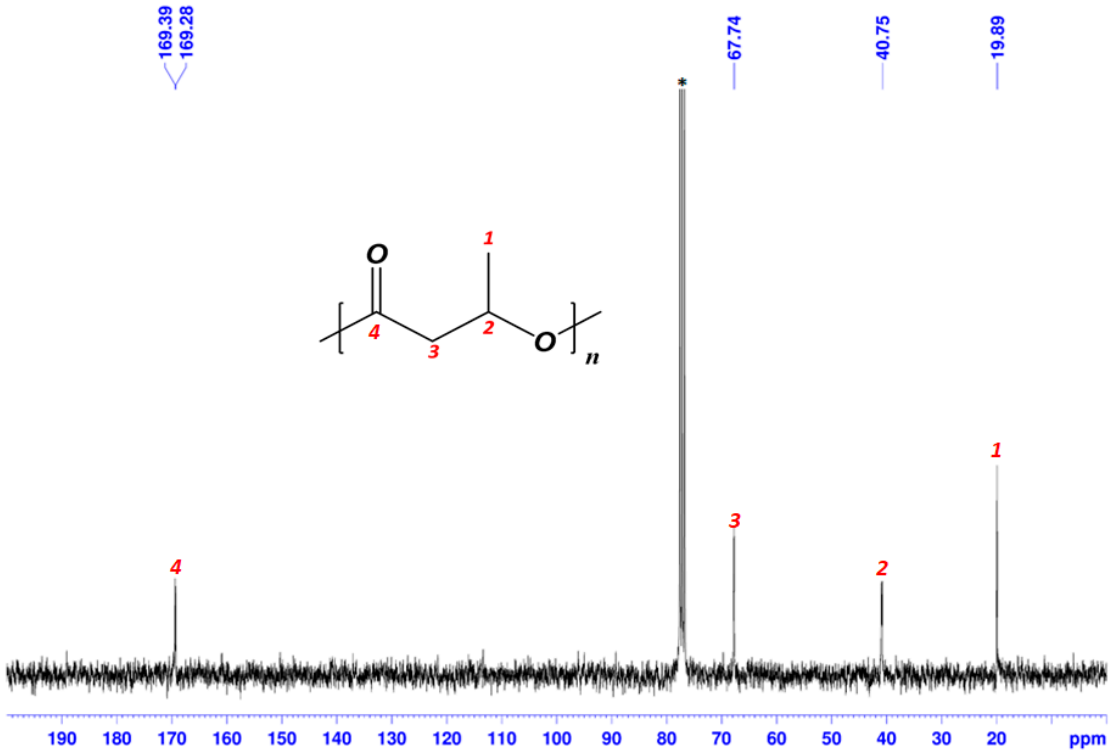

Figure S15: ^{13}C NMR of PHB obtained using (NSO)$AlMe_2$/iPrOH as catalyst (**Run 5, Table 2**), (100.62 MHz, *$CDCl_3$, 25°C).

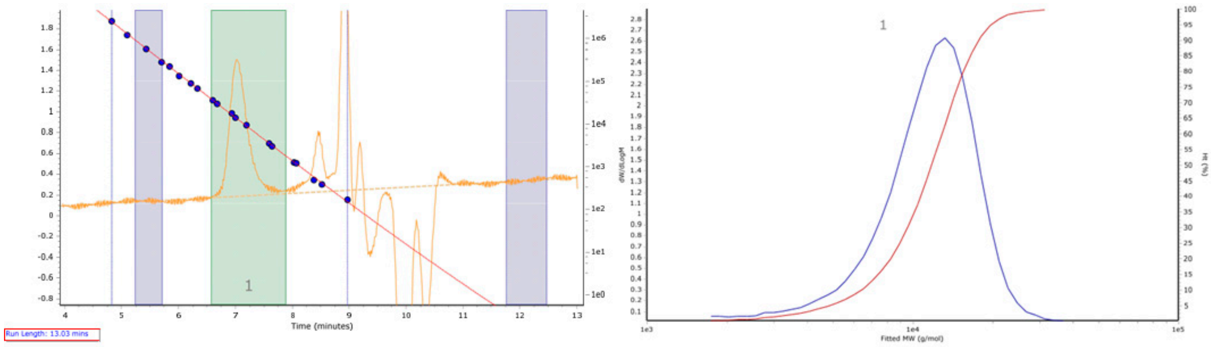

Figure S16: SEC of PLA obtained using (NSO)$AlMe_2$/iPrOH as catalyst **(Run 1**, **Table 1**).

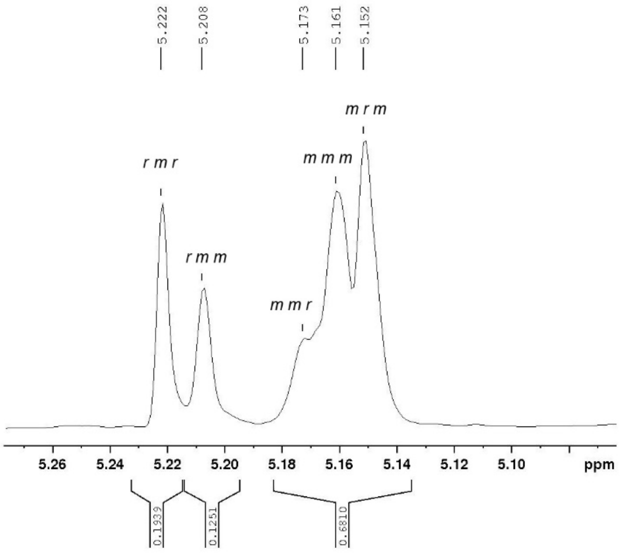

Figure S17. Methine region of the homonuclear decoupled $^{1}H[^{1}H]$ NMR spectrum for *rac*-PLA obtained by (NSO)$AlMe_2$/iPrOH as catalyst (**Run 2**, **Table 1**). The probability of racemic linkage was calculated using the formula: $P_r = (2[rmr])^{1/2}$

Cartesian coordinates and free energies of all the structures optimized in the computational analysis.
Free energies are computed at BP86-D3/TZVP/CPCM//BP86/6-31G(d)

L-lactide

O	1.94033600	1.71471500	-0.41302000
C	1.04614200	0.96641700	-0.06795800
C	-1.04616400	-0.96640700	-0.06792200
O	-1.94029400	-1.71468200	-0.41311400
O	-0.25939800	1.36927300	-0.11436500
O	0.25941400	-1.36929800	-0.11418400
C	-1.26428100	0.46474100	0.44546700
H	-1.13795500	0.45717100	1.54932900
C	1.26427900	-0.46470200	0.44543200
H	1.13804400	-0.45697800	1.54933100
C	-2.63211600	1.01743900	0.07562100
H	-3.41749000	0.37316200	0.50048500
H	-2.74455300	2.04029100	0.46949200
H	-2.75458800	1.03535300	-1.01910900
C	2.63208100	-1.01748700	0.07557900
H	3.41751700	-0.37289000	0.49981900
H	2.74471400	-2.04011100	0.46998900
H	2.75420400	-1.03606600	-1.01918500

Free energy : -534.482581 hartrees

ε-Caprolactone

O	1.94033600	1.71471500	-0.41302000
C	1.04614200	0.96641700	-0.06795800
C	-1.04616400	-0.96640700	-0.06792200
O	-1.94029400	-1.71468200	-0.41311400
O	-0.25939800	1.36927300	-0.11436500
O	0.25941400	-1.36929800	-0.11418400
C	-1.26428100	0.46474100	0.44546700
H	-1.13795500	0.45717100	1.54932900
C	1.26427900	-0.46470200	0.44543200
H	1.13804400	-0.45697800	1.54933100
C	-2.63211600	1.01743900	0.07562100
H	-3.41749000	0.37316200	0.50048500
H	-2.74455300	2.04029100	0.46949200
H	-2.75458800	1.03535300	-1.01910900
C	2.63208100	-1.01748700	0.07557900
H	3.41751700	-0.37289000	0.49981900
H	2.74471400	-2.04011100	0.46998900
H	2.75420400	-1.03606600	-1.01918500

Free energy : -385.138123 hartrees

β-butyrolactone

O	-2.24265900	-0.18737400	-0.31773800
C	-1.08236800	-0.01120200	-0.03933600
C	-0.10504800	1.16586500	0.08679600
H	0.09433700	1.69265800	-0.86122700
H	-0.37425800	1.89685900	0.86620500
C	0.91537700	0.07395900	0.47839800
H	1.21594800	0.09654300	1.54003800
O	-0.14128800	-0.97091700	0.31392100
C	2.09506900	-0.17825600	-0.44111800
H	2.84667400	0.62329300	-0.32434000
H	2.57866300	-1.14082700	-0.20200300
H	1.77203100	-0.20439100	-1.49658000

Free energy : -306.537698 hartrees

Complex 1

C	-2.37913200	1.83624200	-0.21963900
C	-3.61371000	1.90115500	0.43744000
C	-4.27297000	0.71682400	0.80062000
C	-3.68268500	-0.51837500	0.50561500
C	-2.42546800	-0.60165200	-0.13065200
C	-1.77173300	0.59793600	-0.50452500
N	-0.45677400	0.60867700	-1.14751700
S	-1.70770400	-2.21463700	-0.46628900
C	-1.22365400	-2.73456300	1.24316700
C	-0.32828700	-1.70952000	1.97190400
C	0.17930200	-2.30954100	3.29396200
O	0.76238500	-1.35822100	1.15638800
Al	1.07303800	0.09188300	0.23240900
H	-1.86499600	2.76260400	-0.50394600
H	-4.06051100	2.87680600	0.65574000
H	-5.24567900	0.75383800	1.30253900
H	-4.19390800	-1.45096800	0.76738000
H	-2.12941100	-2.96686100	1.83206500
H	-0.66553900	-3.67403600	1.07496000
H	-0.95629400	-0.82363300	2.21475400
H	0.78632200	-1.55949100	3.82831200
H	-0.65480700	-2.61824800	3.95073700
H	0.81746700	-3.18886600	3.09306800
H	-0.21808700	1.61122000	-1.24163700
C	-0.35867800	0.04200500	-2.57956000
C	0.74791100	0.81385600	-3.30889600
C	-1.68750300	0.11752200	-3.34122800
H	-0.05913400	-1.01093000	-2.45503600
H	1.68806600	0.77332400	-2.73353300

H	0.92115800	0.36060600	-4.30026200
H	0.45033100	1.86842400	-3.47451900
H	-2.48728600	-0.47454200	-2.86782600
H	-2.03967500	1.16119100	-3.43711300
H	-1.53331600	-0.28045700	-4.35947900
O	2.43895200	-0.05847000	-0.85218900
O	0.91628200	1.55206500	1.19141200
C	1.22159900	2.89979000	0.87924100
C	2.74024200	3.11997300	0.78098600
C	0.57879200	3.80697900	1.93861900
H	0.77949500	3.18277800	-0.11541900
H	3.17464700	2.44175200	0.02659100
H	2.98054000	4.16384800	0.50508800
H	3.21149000	2.89857200	1.75595300
H	-0.50965200	3.62995000	1.99021800
H	1.00657100	3.57920900	2.93148100
H	0.75507000	4.87550300	1.71645500
C	3.53810400	-0.97083500	-0.82505800
C	3.09479100	-2.37109500	-1.27769000
C	4.20270700	-1.00421500	0.56014400
H	4.28126200	-0.58721900	-1.55718900
H	2.62700800	-2.32296500	-2.27722200
H	3.95411000	-3.06552100	-1.33267100
H	2.35510900	-2.77830400	-0.56566900
H	4.51499100	0.01050000	0.86325200
H	3.49061500	-1.39114600	1.31226700
H	5.09649700	-1.65587000	0.56101300

Free energy : -1626.474684 hartrees

ROP process with *L*-LA

int0

C	1.04463000	-2.89519900	0.02230300
C	1.83385000	-3.86815600	0.64898000
C	3.22192000	-3.68846000	0.75095200
C	3.80576000	-2.52977900	0.22407300
C	3.02397800	-1.52550600	-0.38639600
C	1.62424400	-1.71835600	-0.49266700
N	0.73995600	-0.72760800	-1.11701700
S	3.82835000	-0.04751100	-1.01957200
C	4.28776200	0.78852500	0.56415000
C	3.12491000	0.88303900	1.57422700
C	3.57285600	1.72129700	2.78508500
O	1.98793000	1.46110400	0.98987100
Al	0.53566500	0.93288400	0.15307000
H	-0.03660900	-3.03909400	-0.07771600
H	1.36147700	-4.77415400	1.04386700
H	3.84895500	-4.45118100	1.22511500
H	4.89005300	-2.38434700	0.27660200

H	5.15891600	0.28095700	1.01649000
H	4.59943100	1.79998400	0.24486300
H	2.91373500	-0.15139200	1.92816700
H	2.77248100	1.74558700	3.54422900
H	4.48295700	1.30442900	3.25405300
H	3.77951700	2.76044900	2.47240200
H	-0.21534500	-1.13597500	-1.04525200
C	0.95189900	-0.52141400	-2.63400900
C	-0.30968600	0.11445300	-3.22671800
C	1.29173500	-1.83961900	-3.34368800
H	1.79729600	0.17969300	-2.71939800
H	-0.57265800	1.03789600	-2.68645100
H	-0.12180400	0.35959200	-4.28696100
H	-1.16236300	-0.58501300	-3.18059600
H	2.22770500	-2.29421300	-2.97980800
H	0.47571600	-2.57561600	-3.22519800
H	1.41172700	-1.64001400	-4.42297200
O	-0.13579400	2.21629600	-0.83676300
O	-0.69833200	0.13240800	1.15510700
C	-0.90135600	0.22135500	2.56486700
C	-0.84866900	1.66998200	3.07703700
C	0.07309600	-0.69095300	3.32542400
H	-1.93086100	-0.16153900	2.76491600
H	-1.57626200	2.30295800	2.53852600
H	-1.08193200	1.71962200	4.15690400
H	0.15876200	2.09470100	2.91753400
H	0.03947300	-1.71583300	2.91722800
H	1.10691600	-0.31629000	3.22368100
H	-0.17444500	-0.73244900	4.40250000
C	0.32554500	3.53704200	-1.12668300
C	1.65323000	3.51218300	-1.90012500
C	0.42878100	4.38429500	0.15115300
H	-0.44504900	3.99749100	-1.78302300
H	1.55117600	2.92733400	-2.83133600
H	1.97759400	4.53445700	-2.17054500
H	2.43946500	3.04518800	-1.28057400
H	-0.53750000	4.39245900	0.68561100
H	1.19622400	3.96028600	0.82275800
H	0.70219800	5.42994400	-0.08414000
O	-2.00588300	-2.18304600	-1.03440600
C	-2.79234700	-1.37561300	-0.54360400
C	-4.97032300	0.31502000	0.19766800
O	-6.00031800	0.95948200	0.23902200
O	-2.93625700	-0.15127400	-1.08652200
O	-4.96240200	-1.00500100	0.57106500
C	-3.61614600	0.86116100	-0.26054500
H	-2.95880800	1.02289000	0.61694300
C	-3.67023800	-1.67584100	0.67259300
H	-3.13458800	-1.27035700	1.55573100
C	-3.71636900	2.12973900	-1.08806700
H	-4.23040800	2.90536200	-0.49858200

H	-2.69908100	2.46973100	-1.33866600
H	-4.29831500	1.95298000	-2.00733400
C	-3.94838100	-3.16235300	0.85562700
H	-2.99951500	-3.70816300	0.97158100
H	-4.56836700	-3.31647800	1.75315800
H	-4.48043900	-3.56673700	-0.02074900

Free energy : -2160.964207 hartrees

TS_{0-1}

Imaginary Frequency at: -120 cm^{-1}

C	-2.97547900	-0.03111000	-2.38847700
C	-4.28392300	0.34973000	-2.71138300
C	-5.33007300	0.07011000	-1.82037600
C	-5.04804400	-0.58833100	-0.61637800
C	-3.73413700	-0.96429500	-0.26353900
C	-2.67992500	-0.68009200	-1.17236600
N	-1.28757100	-1.02394000	-0.91050500
S	-3.48310900	-1.84427800	1.28807900
C	-3.37018700	-0.45846600	2.51095700
C	-2.37592100	0.65436000	2.13369700
C	-2.30282400	1.67584700	3.28526900
O	-1.09720200	0.11532600	1.86707000
Al	-0.22710400	0.41630800	0.34381100
H	-2.15930200	0.18175800	-3.08986500
H	-4.48182600	0.85339900	-3.66369300
H	-6.35982700	0.35256800	-2.06406100
H	-5.85545000	-0.82997100	0.08289000
H	-4.37831700	-0.03928200	2.68244900
H	-3.04164700	-0.96783100	3.43616600
H	-2.78051900	1.18297100	1.24424100
H	-1.65802500	2.51916400	2.98585700
H	-3.30033600	2.07882000	3.54245900
H	-1.86979900	1.20612400	4.18746800
H	-0.75854000	-0.72982500	-1.74609800
C	-1.03247200	-2.54245800	-0.81177600
C	-0.08588200	-2.88323900	0.33733800
C	-0.53078800	-3.04970500	-2.17145100
H	-2.01502900	-2.99067800	-0.59763000
H	-0.47709200	-2.47635700	1.28328100
H	-0.00508700	-3.98135800	0.43147600
H	0.92294700	-2.47742300	0.15627900
H	-1.24609100	-2.81319600	-2.98033400
H	0.44857800	-2.60018600	-2.41610800
H	-0.40405700	-4.14629800	-2.14499200
O	1.51551200	0.10107600	0.93059900
O	-0.80339900	1.99037600	-0.17405100
C	-0.41569800	2.98237800	-1.10459900
C	0.38466900	4.08770300	-0.39216000
C	-1.66834900	3.56797400	-1.77700300

H	0.22982500	2.53709000	-1.89582400
H	1.27958000	3.66936500	0.10386400
H	0.71375900	4.87287600	-1.09835300
H	-0.23764100	4.55877300	0.39015600
H	-2.25096700	2.77201600	-2.27121700
H	-2.31837400	4.04016500	-1.01796500
H	-1.39983000	4.33097200	-2.53122200
C	2.10377900	0.22099700	2.25355600
C	1.66978900	-0.95315300	3.13846700
C	1.76819000	1.58037300	2.87653400
H	3.19949000	0.17241900	2.10100700
H	1.95341700	-1.91887400	2.68286400
H	2.15287100	-0.88703900	4.13058200
H	0.57468900	-0.92960000	3.26405200
H	2.09937200	2.40483300	2.22134600
H	0.67906600	1.66178400	3.02630600
H	2.27193900	1.69457500	3.85376800
O	1.04586000	0.18346300	-1.39769900
C	2.20025100	0.02654200	-0.89562900
C	4.88085400	-0.31615200	0.02223800
O	5.97614100	-0.49116800	0.52772500
O	2.74827100	-1.21525600	-1.02425800
O	4.41866300	0.94654100	-0.17769300
C	3.97927800	-1.51395400	-0.30556200
H	3.67165900	-1.92909200	0.67409700
C	3.18619800	1.19026100	-0.91974600
H	2.71531600	2.04543000	-0.41420800
C	4.73183600	-2.57722700	-1.10580500
H	5.64203500	-2.86542400	-0.55830100
H	4.09212000	-3.46357500	-1.24597200
H	5.02090300	-2.18873100	-2.09698900
C	3.54460600	1.55052300	-2.36786000
H	2.63872700	1.86624700	-2.91017200
H	4.27356400	2.37727900	-2.36895400
H	3.98773800	0.68558400	-2.89114300

Free energy : -2160.931935 hartrees

int1

C	-3.44300200	-0.55713500	-2.11257200
C	-4.75916600	-0.15332300	-2.36786800
C	-5.67692100	-0.05913500	-1.31170400
C	-5.25955600	-0.37144000	-0.01124600
C	-3.93242600	-0.76110000	0.26854700
C	-3.00848700	-0.85781400	-0.80609800
N	-1.61842400	-1.24113400	-0.61458800
S	-3.50095200	-1.18813000	1.96439700
C	-3.09065200	0.45855800	2.70096600
C	-2.08580600	1.30730700	1.90316600
C	-1.81064200	2.61557000	2.66808300

O	-0.87185300	0.60031900	1.68815400
Al	-0.26752300	0.35842300	0.04294200
H	-2.73029800	-0.63928800	-2.94219100
H	-5.06529200	0.07614400	-3.39421300
H	-6.71226200	0.24526500	-1.49862800
H	-5.96675100	-0.32106600	0.82337500
H	-4.02443300	1.02619100	2.86600300
H	-2.66562500	0.19446800	3.68737300
H	-2.56183100	1.57994100	0.93700700
H	-1.16512500	3.27077600	2.05902400
H	-2.74457800	3.16476400	2.88922500
H	-1.29438000	2.40227500	3.62191100
H	-1.19566200	-1.23466400	-1.55618400
C	-1.43357700	-2.69028500	-0.11031500
C	-0.42275100	-2.79425000	1.03349000
C	-1.06475300	-3.58236500	-1.30373200
H	-2.42312800	-2.98861800	0.27187300
H	-0.67194900	-2.07162700	1.82739700
H	-0.47227700	-3.81118800	1.46379100
H	0.60535500	-2.61372600	0.68234000
H	-1.82990100	-3.53482100	-2.10045000
H	-0.08797300	-3.27775300	-1.72199000
H	-0.97985300	-4.63498000	-0.98140900
O	1.74442700	0.80448800	0.55815600
O	-0.86824100	1.62347200	-1.01132000
C	-0.32338300	2.28368400	-2.14079100
C	0.45439500	3.53897600	-1.70890600
C	-1.45858200	2.64824000	-3.11071000
H	0.38245900	1.60733500	-2.68048500
H	1.27250200	3.27109100	-1.01783700
H	0.88842700	4.06680700	-2.57876500
H	-0.22136200	4.23551100	-1.17997100
H	-2.01032400	1.74484400	-3.42260700
H	-2.17571500	3.32577900	-2.61293800
H	-1.06897100	3.15117800	-4.01487900
C	2.50030500	1.17911500	1.76793500
C	2.12081400	0.27819900	2.94508100
C	2.20956000	2.65603700	2.02762300
H	3.56899100	1.06817600	1.51576400
H	2.35793200	-0.77694400	2.73133800
H	2.67354600	0.58846000	3.85047800
H	1.03694400	0.36005800	3.13191100
H	2.50809000	3.27688600	1.16664400
H	1.13414700	2.80280700	2.21908400
H	2.77234800	2.99679100	2.91474600
O	0.97914400	-0.68883000	-0.85664300
C	2.17993600	-0.27985100	-0.38869400
C	5.04802000	-0.18209500	-0.18329400
O	6.23064500	-0.02010300	0.06704400
O	2.82516000	-1.34633200	0.27096900
O	4.39598000	0.68666200	-1.00357400

C	4.25851900	-1.31492400	0.48940200
H	4.41948100	-1.15589400	1.57438400
C	3.06414400	0.32750100	-1.49772600
H	2.62096300	1.29451100	-1.78116900
C	4.83447100	-2.68744600	0.11673900
H	5.90628800	-2.71630500	0.37006000
H	4.30554000	-3.47319500	0.68058700
H	4.71669500	-2.89201400	-0.96083600
C	3.19410000	-0.57665000	-2.72321600
H	2.20707600	-0.71068700	-3.19271200
H	3.88192600	-0.11558300	-3.45142800
H	3.58152400	-1.57282300	-2.44934400

Free energy : -2160.935723 hartrees

int2

C	-4.19728300	0.87065000	1.22387800
C	-5.49347100	0.34653700	1.13196700
C	-5.89302100	-0.33117300	-0.03052900
C	-4.98288200	-0.49682000	-1.08338700
C	-3.66537600	-0.00311900	-0.99060500
C	-3.27647400	0.70388200	0.17154500
N	-1.93945400	1.26371900	0.30812900
S	-2.47830400	-0.32209000	-2.29977400
C	-2.21139300	-2.13467300	-2.01398200
C	-1.86523500	-2.48119700	-0.54933800
C	-1.58932400	-3.99088300	-0.43369400
O	-0.74797400	-1.76688000	-0.10118500
Al	-0.35772800	-0.13506200	0.39412100
H	-3.88755700	1.41159900	2.12587300
H	-6.19201200	0.47953200	1.96487300
H	-6.90976000	-0.72876400	-0.11856500
H	-5.28201400	-1.02452800	-1.99533900
H	-3.10090600	-2.69416100	-2.35540600
H	-1.36185800	-2.38549600	-2.67461500
H	-2.76075300	-2.24813400	0.06925400
H	-1.37208800	-4.24620100	0.61719000
H	-2.45583900	-4.59034700	-0.76751300
H	-0.70977300	-4.26859200	-1.04186800
H	-1.81677200	1.49338400	1.31246200
C	-1.66123700	2.55060500	-0.48764500
C	-0.64409300	3.37867100	0.30667100
C	-2.92950000	3.36275500	-0.78126700
H	-1.20513000	2.21568600	-1.43391800
H	0.26361700	2.79546700	0.52556500
H	-0.35040500	4.26306300	-0.28453500
H	-1.08459400	3.74023200	1.25564000
H	-3.66190100	2.80453000	-1.38702800
H	-3.42799400	3.68881400	0.14984000
H	-2.64560500	4.26785900	-1.34634200

O	2.95080600	1.27939000	0.11304700
O	-0.39340500	0.22148000	2.12460900
C	0.30148700	-0.36538100	3.22087900
C	-0.41068800	-1.64797000	3.67895500
C	0.39876000	0.67158200	4.34872800
H	1.33331000	-0.62927700	2.90436700
H	-0.47880200	-2.36416500	2.84179400
H	0.12579000	-2.13205500	4.51660200
H	-1.43793500	-1.41329900	4.01474000
H	0.92237400	1.57686900	3.99566800
H	-0.61310400	0.96621800	4.68439800
H	0.94782600	0.26750200	5.21884500
C	3.69576900	2.25132600	-0.66936800
C	4.66135800	2.90183000	0.32397200
C	2.78413800	3.28042700	-1.34971000
H	4.29354000	1.71682700	-1.43397300
H	5.32017500	2.14352400	0.77917000
H	5.29070800	3.65077200	-0.18800300
H	4.10077600	3.40704700	1.13016000
H	2.03326600	2.79721800	-1.99699600
H	2.24606400	3.87593200	-0.59193100
H	3.38728400	3.96927100	-1.96895300
O	0.82481500	0.71603600	-0.67430100
C	2.12086400	0.30855600	-0.52666600
C	4.37826000	-1.35253100	-0.41965800
O	5.49142200	-1.82957400	-0.26969000
O	2.07556200	-0.80185500	0.39432600
O	4.05370300	-0.77663100	-1.61490900
C	3.36316500	-1.35580000	0.73698600
H	3.82034600	-0.72740800	1.52588800
C	2.71187500	-0.23162600	-1.85120600
H	2.88669500	0.60100400	-2.55316600
C	3.14590300	-2.77636100	1.26316500
H	4.11613600	-3.20855900	1.55389000
H	2.47847900	-2.75213700	2.14033000
H	2.68402000	-3.41414400	0.49102800
C	1.83899100	-1.28556500	-2.52719800
H	0.91769300	-0.81449100	-2.90566300
H	2.39062400	-1.72955800	-3.37309400
H	1.55258800	-2.07588600	-1.81489200

Free energy : -2160.944050 hartrees

TS$_{2\text{-}3}$

Imaginary Frequency at: -91 cm^{-1}

C	-2.65504000	2.09437600	-1.45752200
C	-3.84410300	2.80832500	-1.27195100
C	-4.98952600	2.14938200	-0.80042600
C	-4.92134600	0.78024900	-0.51373300
C	-3.72638500	0.04649200	-0.67702700

C	-2.57448900	0.71659500	-1.16840900
N	-1.29812400	0.04926500	-1.38686500
S	-3.74428000	-1.71219400	-0.28664100
C	-3.42319600	-1.69958500	1.53477400
C	-2.24349400	-0.81988500	1.98310500
C	-2.08023600	-0.94921800	3.51130900
O	-1.04559700	-1.19081700	1.32967400
Al	-0.02799200	-0.05845200	0.42069000
H	-1.75833400	2.61275700	-1.81489400
H	-3.87399700	3.87832300	-1.50531100
H	-5.92950200	2.69442800	-0.66157800
H	-5.80595300	0.24517200	-0.15245700
H	-4.34576000	-1.40306400	2.06650200
H	-3.21115300	-2.76003400	1.76514300
H	-2.50731900	0.23711100	1.75921900
H	-1.25222100	-0.30477400	3.85319200
H	-2.99968900	-0.64811600	4.04682900
H	-1.83790800	-1.99133500	3.78783100
H	-0.67631400	0.76717800	-1.78913800
C	-1.36586200	-1.07833000	-2.43560900
C	-0.61020400	-2.33134800	-1.99396900
C	-0.89320400	-0.53334100	-3.79091900
H	-2.43383800	-1.33403800	-2.51298800
H	-0.93142800	-2.63659600	-0.98441000
H	-0.83994600	-3.16000500	-2.68801500
H	0.47961500	-2.17590200	-1.99952200
H	-1.49282100	0.34036000	-4.10621700
H	0.17031900	-0.23748000	-3.74482700
H	-0.99593700	-1.31084100	-4.56834600
O	3.04940700	1.34994900	-0.78002200
O	-0.29995600	1.65718000	0.65732200
C	0.30682700	2.56764400	1.56401000
C	-0.68890600	2.94725200	2.67248400
C	0.77399600	3.80853100	0.78558600
H	1.19563200	2.09817400	2.04160700
H	-1.00451400	2.05241600	3.23710000
H	-0.24411800	3.66724400	3.38510900
H	-1.59116300	3.40615100	2.22879100
H	1.47077300	3.51061500	-0.01666100
H	-0.09378000	4.31061000	0.31903100
H	1.27858900	4.53787600	1.44746200
C	4.38858300	1.59605700	-0.20511500
C	4.26353300	1.71402500	1.31235100
C	4.88591400	2.87216700	-0.88065700
H	5.05415200	0.75799500	-0.47649000
H	3.77926000	0.82235000	1.74207100
H	5.26447100	1.83829600	1.76233000
H	3.64738600	2.58985800	1.57707500
H	4.94506200	2.74700700	-1.97502300
H	4.20680700	3.71345000	-0.66078000
H	5.89130300	3.12739300	-0.50310800

O	1.32861800	0.08151500	-1.35333500
C	2.50210100	0.12993900	-0.91730600
C	2.15145400	-2.73035400	0.47185000
O	1.69442700	-3.85259400	0.54820300
O	1.65080500	-0.46164600	1.11255600
O	2.65033500	-2.32125700	-0.76646400
C	2.18136500	-1.68066500	1.59966400
H	3.25840200	-1.49902100	1.83894800
C	3.41192300	-1.10996400	-0.89519100
H	4.13071400	-1.03820100	-0.05943800
C	1.51132300	-2.21266800	2.86839300
H	2.02496200	-3.12426800	3.21876900
H	1.56923800	-1.44086400	3.65440700
H	0.45538800	-2.43969300	2.66348200
C	4.15898300	-1.23689000	-2.23427800
H	4.80663800	-0.36415000	-2.42442100
H	4.78028400	-2.14686100	-2.21963500
H	3.43155600	-1.32072800	-3.05824300

Free energy : -2160.927844 hartrees

int3

C	1.38513100	-2.08496200	-1.80240300
C	2.31187800	-3.13049400	-1.87220400
C	3.66941300	-2.87382200	-1.63182600
C	4.07926900	-1.56661500	-1.34327700
C	3.16347800	-0.49334700	-1.25778100
C	1.78583200	-0.77136300	-1.48932900
N	0.71119100	0.21614100	-1.33231300
S	3.86909600	1.16570300	-1.10600900
C	4.04310600	1.60293500	0.68652800
C	2.86180200	1.33909800	1.64620900
C	3.11575000	2.12032500	2.94852300
O	1.60671000	1.71618400	1.10053600
Al	0.44145300	0.46494600	0.68409200
H	0.32009600	-2.28156500	-1.97532600
H	1.97010000	-4.14130300	-2.11995900
H	4.40834900	-3.68046100	-1.68636000
H	5.14034900	-1.34357100	-1.19107100
H	4.95204100	1.12268800	1.09212600
H	4.23859100	2.68997400	0.61214400
H	2.86804900	0.25198200	1.87639000
H	2.33847300	1.87664100	3.69235500
H	4.10326700	1.88266200	3.38569000
H	3.06931500	3.20592600	2.74876200
H	-0.17542700	-0.24793900	-1.62506300
C	0.79924900	1.44792800	-2.25439800
C	-0.29265900	2.45743200	-1.90530000
C	0.73280800	0.99761200	-3.71918600
H	1.77986700	1.89164300	-2.03728000

H	-0.18363200	2.83387900	-0.87455500
H	-0.19488400	3.32807700	-2.57760500
H	-1.30074400	2.03428100	-2.03804200
H	1.53088100	0.27471900	-3.96286100
H	-0.24491400	0.53753900	-3.94988100
H	0.86081600	1.87403300	-4.37825900
O	-2.66397100	-1.86818700	-0.74176900
O	0.82552900	-1.12741200	1.31367700
C	0.52602400	-1.75875100	2.54944100
C	0.72414100	-0.83232700	3.76153700
C	1.39317000	-3.02123600	2.67038700
H	-0.54253700	-2.07461700	2.55339400
H	0.10047600	0.07551600	3.66838400
H	0.43808000	-1.34307400	4.70003000
H	1.78029500	-0.52069000	3.84981600
H	1.24060500	-3.67927300	1.79765700
H	2.46231400	-2.74325900	2.69868900
H	1.15339700	-3.59003800	3.58770000
C	-2.97178400	-2.35092600	0.62010700
C	-2.04321000	-3.55253100	0.79441300
C	-4.44376800	-2.74235100	0.77101900
H	-2.68665400	-1.54681100	1.32262100
H	-0.99649200	-3.25044900	0.63270700
H	-2.14049400	-3.96705100	1.81273200
H	-2.30396400	-4.34410400	0.06959200
H	-5.12815600	-1.87936200	0.73065400
H	-4.73236300	-3.45690400	-0.02004100
H	-4.59177100	-3.23748900	1.74717400
O	-2.12166900	-0.22538600	-2.12595700
C	-2.77764600	-0.57784100	-1.15012200
C	-2.86284600	2.47523500	0.22120300
O	-2.83715000	3.67944000	0.04766500
O	-1.30072400	0.74885900	0.83765900
O	-3.55657900	1.71585800	-0.70601100
C	-2.14487000	1.74300800	1.37791200
H	-2.93554700	1.23739000	1.98361800
C	-3.86134800	0.31816200	-0.52533500
H	-3.98676900	0.08669600	0.54569900
C	-1.44082800	2.75172800	2.28979800
H	-2.15927100	3.49594200	2.67268500
H	-0.98851600	2.22080900	3.14498600
H	-0.64390500	3.28540100	1.74746600
C	-5.17879100	0.10482800	-1.29351900
H	-5.48226700	-0.95456000	-1.29425000
H	-5.97827200	0.71129000	-0.83734300
H	-5.03851000	0.43170200	-2.33683300

Free energy : -2160.944623 hartrees

int4

C	-1.05798400	-1.83970400	-1.76200800
C	-1.64837200	-2.30125200	-2.94328600
C	-3.02320800	-2.57984200	-2.97422600
C	-3.78621600	-2.39692000	-1.81478300
C	-3.21422400	-1.91650400	-0.61565100
C	-1.82129200	-1.63823800	-0.59522100
N	-1.12613300	-1.07924700	0.56584200
S	-4.26447400	-1.81980600	0.84687300
C	-5.15969400	-0.20941200	0.68231500
C	-4.29186900	1.03300300	0.39189800
C	-5.16082100	2.29371100	0.55213100
O	-3.17629600	1.10878300	1.26044000
Al	-1.54203500	0.94226600	0.64680500
H	0.01214400	-1.60509900	-1.73805900
H	-1.03081500	-2.44606400	-3.83635700
H	-3.49677600	-2.94902300	-3.89032700
H	-4.85502600	-2.63538400	-1.81356800
H	-5.95467900	-0.30330600	-0.07991400
H	-5.64280400	-0.11167900	1.67283200
H	-3.96197500	0.96976800	-0.66910300
H	-4.57787000	3.18789900	0.27344900
H	-6.06553100	2.25448600	-0.08271500
H	-5.47078800	2.40497400	1.60670800
H	-0.12432300	-0.97061600	0.30468500
C	-1.12717700	-1.92222100	1.85022400
C	-0.07232200	-1.38055700	2.82183900
C	-0.88741600	-3.40542700	1.53678800
H	-2.13042400	-1.79311000	2.28251200
H	-0.21986700	-0.30980700	3.03171100
H	-0.14247000	-1.94520700	3.76803300
H	0.94890600	-1.51081300	2.42016200
H	-1.66398200	-3.82673100	0.87697700
H	0.09744900	-3.55897300	1.05689600
H	-0.89590000	-3.98035100	2.47920800
O	5.24569700	-0.62074800	-0.69207100
O	-1.29223500	1.31031100	-1.04828300
C	-0.90490700	2.49576700	-1.72231500
C	-1.58861800	3.74650500	-1.14335500
C	-1.21302500	2.33134800	-3.21838400
H	0.19804900	2.63380000	-1.61740700
H	-1.36028900	3.86168400	-0.06718500
H	-1.25469200	4.66463200	-1.66196900
H	-2.68563500	3.66660900	-1.25329800
H	-0.72466900	1.42258700	-3.60999600
H	-2.30237400	2.22429300	-3.37138600
H	-0.86000600	3.20215900	-3.80099900
C	6.12996400	-1.56865000	0.01457900
C	7.26396800	-1.88529900	-0.95626500
C	5.31208800	-2.79077100	0.43208200
H	6.50775000	-1.04680000	0.91078800
H	7.80736800	-0.96916900	-1.24335000

H	7.98088700	-2.57849000	-0.48245400
H	6.87582300	-2.36339200	-1.87288100
H	4.50361800	-2.49881600	1.12172400
H	4.87026900	-3.28497300	-0.45114700
H	5.96063700	-3.51992800	0.94965000
O	4.61516800	0.32929600	1.30191500
C	4.56188600	0.25983500	0.08444300
C	1.75588900	1.25315500	0.50240800
O	1.55197200	0.07097200	0.22924100
O	-0.32778000	1.59578600	1.75130100
O	2.81150100	1.95060700	-0.00628700
C	0.90876600	2.15602300	1.42279300
H	0.79032000	3.11352500	0.86589400
C	3.73637400	1.19348200	-0.82091000
H	3.16648400	0.57059400	-1.53141100
C	1.71077200	2.45429900	2.70896400
H	2.67728800	2.93136700	2.47898800
H	1.11058700	3.12546300	3.34514200
H	1.89870500	1.52031100	3.26451400
C	4.61831400	2.20767700	-1.55330400
H	5.33454900	1.67902800	-2.20273900
H	3.99332500	2.86963200	-2.17508100
H	5.17824000	2.82698600	-0.83244000

Free energy : -2160.967880 hartrees

ROP process with ε–caprolactone (CL)

int0 $_{CL}$

C	0.52109100	-2.69392300	0.54072000
C	1.20747800	-3.73521600	1.17670600
C	2.58380900	-3.90062800	0.96130100
C	3.24991800	-3.02604400	0.09456800
C	2.58213300	-1.95738300	-0.54330400
C	1.19260200	-1.78643200	-0.30328800
N	0.40174200	-0.67838100	-0.85519200
S	3.51026700	-0.96041100	-1.72411500
C	4.48895100	0.18673700	-0.65430900
C	3.66383700	0.96610800	0.38895400
C	4.54382500	2.05593300	1.02629800
O	2.52386600	1.55467400	-0.20905500
Al	0.89002900	1.06230500	0.20492000
H	-0.55535100	-2.56908800	0.70313000
H	0.65967600	-4.42149200	1.83166700
H	3.13130600	-4.71587300	1.44650100
H	4.31559200	-3.16453700	-0.11674700
H	5.31148400	-0.36713400	-0.16611800
H	4.93020900	0.88331900	-1.39191800
H	3.36748600	0.25138100	1.18714700

H	3.98289800	2.56470100	1.82853000
H	5.46832500	1.63407000	1.46287900
H	4.82231600	2.80978200	0.26772200
H	-0.57484000	-0.83976300	-0.52851100
C	0.29630700	-0.59440000	-2.38942500
C	-0.96579900	0.19937900	-2.74705000
C	0.27759300	-1.98206600	-3.04393900
H	1.19176400	-0.04287600	-2.71527800
H	-0.99494400	1.15009300	-2.18970500
H	-0.96822100	0.41068600	-3.83062500
H	-1.87533000	-0.38331500	-2.51007200
H	1.20423700	-2.55144500	-2.86604200
H	-0.57483400	-2.58406700	-2.67834400
H	0.16301000	-1.86041100	-4.13559400
O	-0.34506800	2.19083300	-0.35287700
O	0.83887000	0.47976800	1.84716400
C	-0.12289500	0.16539800	2.83646900
C	-0.80722400	1.43786100	3.36467800
C	0.57429100	-0.60892600	3.96561900
H	-0.91071000	-0.49743500	2.40796900
H	-1.28005000	1.99300000	2.53573000
H	-1.58175700	1.19653700	4.11688500
H	-0.05844300	2.10049500	3.83625000
H	1.05720300	-1.51592600	3.56332300
H	1.35666400	0.02153700	4.42599400
H	-0.14369500	-0.90818300	4.75148900
C	-0.20250400	3.59799900	-0.58361700
C	0.57946100	3.86199600	-1.88070000
C	0.44657800	4.30627700	0.61579700
H	-1.23186200	3.99964600	-0.70779600
H	0.08716100	3.37346400	-2.74017800
H	0.64344000	4.94570700	-2.09330400
H	1.60375100	3.45878300	-1.78873700
H	-0.12359900	4.11260000	1.54096500
H	1.47886500	3.93620200	0.75528500
H	0.48934200	5.40014400	0.45721200
O	-2.45735500	-1.19428100	0.09237100
C	-3.34711300	-0.47356900	0.54050500
C	-3.50726700	1.00890100	0.33872400
C	-4.51084400	1.17915700	-0.84900200
H	-3.94204600	1.44905500	1.25297100
H	-2.54117200	1.49124800	0.10707000
C	-5.22155300	-1.90381900	0.34571100
C	-5.81681400	0.34178900	-0.80312900
H	-3.97501800	0.94615300	-1.78915200
H	-4.77661600	2.25090100	-0.89928400
C	-5.62761400	-1.19294700	-0.96240400
H	-6.09756900	-2.14789900	0.96737300
H	-4.64241400	-2.82601100	0.16020000
H	-6.37543400	0.55780400	0.12782600
H	-6.45327500	0.69389700	-1.63636400

H	-6.56421300	-1.65217200	-1.33031600
H	-4.86484900	-1.38050700	-1.74266800
O	-4.45716500	-0.98235500	1.17434400

Free energy : -2011.610893 hartrees

$TS_{0\text{-}1CL}$

Imaginary Frequency at: - 154 cm^{-1}

C	1.82690600	-2.75372600	0.04795300
C	2.88620800	-3.43445800	0.66197400
C	4.16496100	-2.85892000	0.68488400
C	4.36828600	-1.61070200	0.08157500
C	3.31369200	-0.90153600	-0.53187000
C	2.01939100	-1.48966300	-0.54263800
N	0.85792500	-0.82469800	-1.12422600
S	3.69559600	0.63692000	-1.39027500
C	3.67505900	1.93487200	-0.07201100
C	2.42268400	1.96157200	0.82606300
C	2.46690900	3.22264200	1.70932400
O	1.23367100	1.94741400	0.05789600
Al	0.06376800	0.61871500	0.22754400
H	0.82493200	-3.19932000	0.03705300
H	2.70920000	-4.41646600	1.11389000
H	5.00303700	-3.38337200	1.15628900
H	5.36647700	-1.16061000	0.06683900
H	4.58841500	1.84955500	0.54441300
H	3.74407600	2.86696600	-0.66475300
H	2.46597300	1.07719900	1.49574600
H	1.62341200	3.20082400	2.42014800
H	3.40621800	3.28851600	2.28978800
H	2.37321700	4.13038900	1.08542600
H	0.04112700	-1.44677900	-0.97711800
C	0.95255900	-0.63046900	-2.64919100
C	0.43869000	0.74241400	-3.07575300
C	0.23894900	-1.79962500	-3.34277700
H	2.02661000	-0.67930200	-2.88498800
H	1.01292800	1.53116700	-2.56340300
H	0.56151900	0.85978700	-4.16742100
H	-0.62764900	0.87264800	-2.82822900
H	0.66690600	-2.77104300	-3.03510200
H	-0.84161400	-1.80452200	-3.10821900
H	0.34654700	-1.71543100	-4.43837800
O	-1.49150400	1.39391400	-0.40194600
O	0.35464900	0.00242000	1.83391000
C	-0.27567000	-0.82229200	2.79632500
C	-1.13244400	0.03438000	3.74454500
C	0.79922400	-1.59458800	3.57863300
H	-0.93808700	-1.55658000	2.28701500
H	-1.90915200	0.58021900	3.18146800
H	-1.62843800	-0.58761100	4.51370200

H	-0.49578700	0.77803100	4.25782900
H	1.42265400	-2.19389800	2.89388600
H	1.46099500	-0.88789500	4.11232900
H	0.34069400	-2.27159500	4.32338800
C	-1.98073800	2.75547100	-0.29207800
C	-1.26125700	3.65022800	-1.30751800
C	-1.85423300	3.27401100	1.14382600
H	-3.05587200	2.69154100	-0.55405900
H	-1.40573000	3.27555200	-2.33567800
H	-1.65622000	4.68166900	-1.25759000
H	-0.18103200	3.66722500	-1.08462000
H	-2.39444900	2.61312600	1.84114800
H	-0.79031500	3.31515200	1.43559700
H	-2.27815800	4.29181600	1.22674500
O	-1.64545600	-0.97972500	-0.10084900
C	-2.57033700	-0.14350200	-0.35201700
C	-3.26957200	-0.08683200	-1.70517600
C	-4.19999100	-1.31655200	-1.86281400
H	-3.86090100	0.84384900	-1.76330400
H	-2.52562800	-0.07720300	-2.51925200
C	-3.96552300	-0.87639400	1.45523700
C	-5.20442100	-1.56774400	-0.71314200
H	-3.57158100	-2.22147500	-1.98072200
H	-4.75813400	-1.19660800	-2.81068100
C	-4.54497000	-2.02709600	0.60965000
H	-4.75298100	-0.40432400	2.06684400
H	-3.16964600	-1.24072200	2.12856100
H	-5.81575500	-0.66067400	-0.53886400
H	-5.90375700	-2.35718800	-1.04764300
H	-5.28360800	-2.56453300	1.23513200
H	-3.74505300	-2.75386000	0.37484600
O	-3.48205500	0.22419100	0.64471700

Free energy : -2011.588834 hartrees

int1$_{CL}$

C	-2.81415300	-1.84763600	-1.41920800
C	-4.08637200	-1.76187400	-1.99888600
C	-5.10941500	-1.06827100	-1.33649100
C	-4.84108900	-0.47146900	-0.09744600
C	-3.56192300	-0.53255100	0.49492700
C	-2.52858900	-1.23605100	-0.18207400
N	-1.17910700	-1.34101700	0.34530500
S	-3.33598800	0.19971000	2.12566600
C	-2.97557200	1.97468500	1.74660000
C	-1.87247000	2.22411300	0.70234800
C	-1.64939200	3.74355100	0.57082400
O	-0.65964400	1.58514600	1.06324800
Al	0.11276300	0.43586700	-0.03660800
H	-2.01668500	-2.39234300	-1.94009700

H	-4.27564800	-2.24752700	-2.96239500
H	-6.11122000	-1.00124400	-1.77403700
H	-5.63249900	0.05741900	0.44407600
H	-3.91090200	2.47853900	1.44199200
H	-2.66696800	2.37885400	2.72900000
H	-2.24249100	1.85256800	-0.27745400
H	-0.92557700	3.94242300	-0.23764100
H	-2.58705200	4.27863100	0.33051600
H	-1.24107300	4.15285100	1.51296100
H	-0.64679100	-1.89567100	-0.34370100
C	-1.08818000	-2.14308000	1.65802000
C	-0.21047700	-1.45516500	2.70446200
C	-0.62045800	-3.56927700	1.33794600
H	-2.11922700	-2.18334800	2.04382500
H	-0.51746000	-0.40343600	2.82430700
H	-0.32878400	-1.96996500	3.67510200
H	0.85009500	-1.49385500	2.41130900
H	-1.29697800	-4.06842500	0.61995000
H	0.40173900	-3.55440900	0.91611700
H	-0.59534100	-4.18022300	2.25745600
O	2.00988000	1.27643300	0.25192700
O	-0.41748500	0.75276300	-1.67466100
C	-0.07914300	0.23813500	-2.95070200
C	1.17852200	0.92260700	-3.51300600
C	-1.27483700	0.43569900	-3.89640500
H	0.13303600	-0.85805500	-2.88310300
H	2.03737900	0.78568400	-2.83444600
H	1.44450000	0.51457600	-4.50657600
H	0.99220500	2.00668500	-3.62390100
H	-2.17997100	-0.04087900	-3.48425900
H	-1.48208900	1.51522600	-4.01275100
H	-1.07100100	0.01011400	-4.89658900
C	2.82159800	2.49511400	0.33040000
C	2.61957300	3.13062800	1.70826400
C	2.41728600	3.41549600	-0.82029600
H	3.86910400	2.17347600	0.19038000
H	2.98252500	2.46871000	2.51251000
H	3.16670200	4.08836200	1.77495100
H	1.54524300	3.31759300	1.87411900
H	2.58943300	2.92527400	-1.79099300
H	1.34767500	3.67581300	-0.74455100
H	3.00684100	4.34925700	-0.78303600
O	1.46131500	-0.83015700	0.12630300
C	2.59555200	-0.07666500	0.24305500
C	3.41554200	-0.32404400	1.52374200
C	3.93955400	-1.76485300	1.67312700
H	4.27139700	0.37715400	1.51564600
H	2.78416600	-0.06869800	2.39422900
C	3.56604000	-1.35061400	-1.61305700
C	4.74872700	-2.31978700	0.48134600
H	3.08316900	-2.44219700	1.85867500

H	4.56527800	-1.80044000	2.58552300
C	3.88035000	-2.59731400	-0.76357400
H	4.38945500	-1.15459700	-2.32234500
H	2.64233000	-1.51270200	-2.20088400
H	5.57684400	-1.62920100	0.22613000
H	5.22031400	-3.26850600	0.80124900
H	4.38366600	-3.32986900	-1.42464400
H	2.93554000	-3.06812700	-0.43458900
O	3.47702300	-0.11271900	-0.88149500

Free energy : -2011.601738 hartrees

int2$_{CL}$

C	2.72781200	-0.05599700	-2.62194600
C	3.77825800	-0.87530000	-3.05594700
C	4.70072500	-1.38175200	-2.12716000
C	4.54436300	-1.08318700	-0.76642100
C	3.46980500	-0.29146200	-0.31205000
C	2.56236600	0.24858800	-1.25615500
N	1.46884700	1.11032800	-0.84922600
S	3.22139000	-0.02820900	1.44740600
C	2.58116300	-1.70386700	1.92450400
C	1.54945700	-2.29699600	0.94118500
C	1.10320400	-3.67528900	1.46804900
O	0.42669100	-1.48587700	0.75191400
Al	-0.11674100	0.13391500	0.34628500
H	2.01931200	0.35644000	-3.35041600
H	3.88265500	-1.10267300	-4.12235000
H	5.53777000	-2.00538900	-2.45900300
H	5.25393000	-1.47386200	-0.02916000
H	3.43294500	-2.39689000	2.04792900
H	2.11676200	-1.54058000	2.91435200
H	2.07456600	-2.46125900	-0.02607300
H	0.40036900	-4.13457000	0.75227800
H	1.96176300	-4.35923000	1.59832300
H	0.58458400	-3.56811400	2.43778300
H	0.85372700	1.22107300	-1.67293900
C	1.87915100	2.52099300	-0.41754700
C	0.65702300	3.43992000	-0.52341600
C	3.05665600	3.07125000	-1.23684900
H	2.17084900	2.42752400	0.64164700
H	-0.16287500	3.07470200	0.11364800
H	0.93435800	4.45382800	-0.18529200
H	0.30918300	3.52117500	-1.57089800
H	3.96192800	2.44821100	-1.15070900
H	2.79690900	3.15656900	-2.30874400
H	3.30617300	4.08295700	-0.87149200
O	-0.32001400	1.34415300	1.60326400
C	-0.04125500	1.28590000	2.99443000
C	-0.95346800	0.27345400	3.70916000

C	-0.19997000	2.69582200	3.58287100
H	1.01525000	0.96668000	3.15009700
H	-0.82559400	-0.73106500	3.26792100
H	-0.72026600	0.21243800	4.78906800
H	-2.01259000	0.57175000	3.59935100
H	0.47414000	3.40554600	3.07223400
H	-1.23831400	3.04901800	3.44425200
H	0.03359300	2.70947000	4.66336000
C	-3.41571900	1.87600600	-0.78922000
C	-4.81656300	2.05999300	-0.20012300
C	-3.10010200	2.89825500	-1.88583800
H	-2.66184800	1.93655400	0.01544900
H	-5.00781800	1.32519200	0.60054800
H	-4.92730800	3.07294400	0.22709300
H	-5.58621500	1.92958700	-0.98233600
H	-2.09392300	2.71338700	-2.29476500
H	-3.83393000	2.81806000	-2.70834700
H	-3.13716200	3.92841400	-1.48648000
O	-1.02661200	0.41631000	-1.21750900
C	-2.23495300	-0.23714000	-1.09424200
C	-2.31523600	-1.48561600	-1.99622700
C	-3.17544600	-1.42385300	0.95764400
C	-3.61461100	-2.30762500	-1.88178100
H	-1.42349600	-2.10120200	-1.78091400
H	-2.20101600	-1.10123000	-3.02561500
C	-2.98451200	-2.90194600	0.59179700
H	-4.17970600	-1.04727100	0.68243300
H	-3.03498500	-1.26995000	2.04027800
C	-3.63156700	-3.35256200	-0.74116500
H	-3.79667600	-2.83501100	-2.83717200
H	-4.45408900	-1.59871300	-1.76578200
H	-1.89321900	-3.07475800	0.57816200
H	-3.38665500	-3.52476300	1.41466600
H	-3.11556400	-4.27313000	-1.07671300
H	-4.68364600	-3.64737200	-0.56015200
O	-2.18705700	-0.56310100	0.35260200
O	-3.36924400	0.54078200	-1.37558000

Free energy : -2011.608713 hartrees

$TS_{2\text{-}3CL}$

Imaginary Frequency at: - 130 cm^{-1}

C	3.76266100	1.31463800	1.03126100
C	5.00508800	1.44283100	0.39882900
C	5.48754900	0.40814000	-0.41940500
C	4.69618800	-0.73174600	-0.61768700
C	3.42082200	-0.85069400	-0.02891600
C	2.94678400	0.17367200	0.83688500
N	1.67566300	0.09262500	1.46509600
S	2.37119700	-2.24943000	-0.44928400

C	1.98816200	-1.81041300	-2.20709100
C	1.35946900	-0.41662900	-2.41126100
C	1.17804300	-0.15960600	-3.91987300
O	0.10982300	-0.32109800	-1.77297400
Al	-0.60465900	0.43354800	-0.37516400
H	3.40558400	2.10790900	1.70006400
H	5.60795600	2.34234500	0.56782600
H	6.47054000	0.48605000	-0.89634400
H	5.05427100	-1.54704600	-1.25647300
H	2.90989900	-1.90581800	-2.80941700
H	1.27662800	-2.58955700	-2.53710500
H	2.07403700	0.33649200	-2.01396200
H	0.74970800	0.84492600	-4.07956300
H	2.13901500	-0.21813700	-4.46287800
H	0.47991500	-0.89896000	-4.35242400
H	1.41084800	1.01896800	1.81994900
C	1.44327300	-0.94126400	2.53774900
C	0.45804000	-0.35017100	3.55566300
C	2.72871700	-1.40940900	3.24168300
H	0.96887800	-1.80839800	2.04496500
H	-0.44957200	0.01614400	3.05069800
H	0.15918800	-1.11966600	4.28874000
H	0.92367700	0.48459400	4.11501100
H	3.44938500	-1.86306700	2.54093000
H	3.23363900	-0.56912900	3.75352500
H	2.47467100	-2.17006900	4.00185000
O	-0.21023000	2.04713600	0.19699700
C	0.61887800	3.04919500	-0.38548500
C	0.75508500	4.20583000	0.61547000
C	0.04693800	3.53731600	-1.72723900
H	1.63533300	2.63510300	-0.57345600
H	1.16820900	3.84457800	1.57422500
H	1.42038800	4.99819900	0.22687800
H	-0.23619600	4.64961100	0.82107400
H	-0.04476500	2.69779900	-2.44017000
H	-0.95649600	3.97665800	-1.57717600
H	0.69628100	4.30553900	-2.18695500
C	-2.58954300	-2.74674400	-0.47918600
C	-3.60551200	-3.16306600	-1.53952300
C	-2.12545400	-3.89339100	0.41784100
H	-1.72422100	-2.25149400	-0.94869800
H	-3.91333900	-2.29370200	-2.14371600
H	-3.15709300	-3.91370200	-2.21395800
H	-4.50310700	-3.60818300	-1.07435700
H	-1.39690200	-3.53164600	1.16095700
H	-2.98251800	-4.34947600	0.94505700
H	-1.63786000	-4.67525100	-0.19127700
O	-1.27921400	-0.74394900	0.89595600
C	-2.58628600	-0.66769600	0.76634000
C	-3.39511500	0.18616300	1.73258000
C	-3.30157300	1.39811000	-1.33833600

C	-4.78586800	0.61250700	1.20912300
H	-2.77453100	1.05477600	2.00964000
H	-3.49718900	-0.43478200	2.64438700
C	-3.72363900	2.46714200	-0.31931200
H	-4.18577500	0.88387300	-1.76880300
H	-2.76437600	1.87643600	-2.18073000
C	-4.85499300	2.05088300	0.64884700
H	-5.52856000	0.52481300	2.02328200
H	-5.10205700	-0.11290800	0.43864700
H	-2.80972900	2.75367100	0.23348300
H	-4.05029300	3.37347900	-0.86672500
H	-4.87070500	2.77033000	1.49109200
H	-5.83240300	2.16308600	0.13921900
O	-2.42547500	0.41378200	-0.78663800
O	-3.27801300	-1.74732200	0.36653100

Free energy : -2011.603078 hartrees

int3$_{CL}$

C	-1.14368800	-2.06683100	-1.49424500
C	-1.90758000	-3.15646500	-1.05272200
C	-1.39750800	-4.02574200	-0.07501600
C	-0.13571300	-3.77859100	0.48350800
C	0.63019800	-2.67118200	0.06884700
C	0.13225900	-1.82908800	-0.95359800
N	0.93895700	-0.72070200	-1.44987500
S	2.19142500	-2.27863400	0.86734400
C	1.58257500	-1.67199800	2.51148300
C	0.44337000	-0.63370700	2.40466600
C	0.14232600	-0.06676400	3.80364000
O	0.72657900	0.41617400	1.52149200
Al	1.57961300	0.63783000	-0.00279700
H	-1.55357700	-1.37729900	-2.23879400
H	-2.89971500	-3.32586800	-1.48361100
H	-1.98275100	-4.88944300	0.25871000
H	0.26694300	-4.43883700	1.25906100
H	1.27410100	-2.53226500	3.13208600
H	2.48332200	-1.21832600	2.96484400
H	-0.46206100	-1.18052300	2.05596400
H	-0.68977200	0.65394500	3.73510300
H	-0.14254600	-0.86366800	4.51490900
H	1.02430400	0.46654600	4.20168200
H	0.31190700	-0.05824000	-1.94244200
C	2.01772700	-1.14212000	-2.46003200
C	2.66225800	0.10985400	-3.06660500
C	1.45608300	-2.06958600	-3.54607100
H	2.76833800	-1.68364000	-1.85699500
H	3.16949900	0.71209600	-2.29638500
H	3.41090000	-0.20434000	-3.81524000
H	1.91058300	0.73695200	-3.57919000

H	1.01137400	-2.98855500	-3.13112400
H	0.68855000	-1.55404300	-4.15332300
H	2.27503600	-2.36448700	-4.22509400
O	3.31341900	0.89955700	-0.01995900
C	4.32061200	0.69176800	0.95680600
C	4.21913900	1.74333800	2.07442300
C	5.69117200	0.72932400	0.26428500
H	4.20293900	-0.31806500	1.41757600
H	3.22551700	1.69715700	2.55456100
H	4.99193900	1.58769600	2.85067000
H	4.34907000	2.75666400	1.65214500
H	5.74524100	-0.04039700	-0.52526100
H	5.84971500	1.71648900	-0.20699900
H	6.51045800	0.54921200	0.98427000
C	-5.39320600	-0.13721700	0.25660100
C	-6.76460600	0.36614400	0.70240800
C	-4.93951900	-1.40296300	0.98691400
H	-5.36785900	-0.30497400	-0.83436800
H	-7.03813500	1.29081700	0.16656500
H	-7.53516300	-0.39714600	0.49412700
H	-6.77172200	0.57783600	1.78636800
H	-3.94440900	-1.72044500	0.63325900
H	-4.88917600	-1.22649500	2.07613800
H	-5.65223200	-2.22729900	0.80267000
O	-3.20732500	0.38209700	-1.29304100
C	-3.38710800	1.08906900	-0.30414100
C	-2.48574100	2.22767400	0.13304900
C	1.11836600	3.28418200	-0.85260000
C	-2.08041900	3.12684600	-1.05246600
H	-1.57687000	1.77396400	0.57527200
H	-2.98937100	2.80629400	0.92748400
C	0.12021000	4.06303700	0.02488400
H	1.12950900	3.73463900	-1.87073700
H	2.14187400	3.41700300	-0.43956300
C	-1.24503500	4.35242000	-0.63240500
H	-2.99553100	3.48225000	-1.56630600
H	-1.51830100	2.51458100	-1.77646500
H	-0.00871200	3.51025500	0.97579300
H	0.58222600	5.03580300	0.29185400
H	-1.84749300	4.98006500	0.05629600
H	-1.07248700	4.97420800	-1.53460500
O	0.80232300	1.90259900	-0.96735500
O	-4.44661200	0.94491700	0.54979000

Free energy : -2011.629521 hartrees

ROP process with β–Butyrolactone (BL)

int0$_{BL}$

C	1.08395800	-2.70638600	0.39126100
C	1.96477800	-3.48774600	1.14835800
C	3.32122900	-3.13761900	1.22710600
C	3.77930200	-2.01284200	0.53120300
C	2.90534400	-1.19964900	-0.22407200
C	1.53231600	-1.55440100	-0.28579700
N	0.52104700	-0.75875300	-0.99546800
S	3.60979400	0.16931100	-1.15978000
C	3.88304500	1.46553400	0.13000800
C	2.64641600	1.79139800	0.99212400
C	2.93057900	3.04058300	1.84440200
O	1.51263700	2.01357200	0.17576300
Al	0.11338800	0.95157700	0.15840300
H	0.02479900	-2.98018500	0.32924500
H	1.58801000	-4.37575100	1.66743500
H	4.02175300	-3.74585700	1.80943900
H	4.84129600	-1.74586500	0.55112200
H	4.74113300	1.18511800	0.76779300
H	4.17156100	2.34785100	-0.47161200
H	2.47709200	0.93542100	1.68092100
H	2.07872800	3.23236600	2.51866400
H	3.84077800	2.91818700	2.46045700
H	3.06067900	3.92305800	1.19199900
H	-0.37107100	-1.28677700	-0.88458200
C	0.70469000	-0.62050300	-2.51804700
C	-0.64586000	-0.24354700	-3.13739600
C	1.26174800	-1.90344100	-3.14900000
H	1.42391300	0.20180700	-2.65403300
H	-1.09090700	0.61321700	-2.60491400
H	-0.49968100	0.02324500	-4.19859200
H	-1.34700900	-1.09751200	-3.09362800
H	2.26394500	-2.16287300	-2.77085300
H	0.58897400	-2.76187900	-2.96752800
H	1.34035700	-1.76003800	-4.24114500
O	-1.28881700	1.67014200	-0.64539200
O	-0.10997800	0.19757700	1.71118400
C	-1.12265800	-0.38890500	2.50481000
C	-2.13256500	0.67020700	2.97889100
C	-0.46170500	-1.10301500	3.69392200
H	-1.67602800	-1.15529400	1.91414800
H	-2.58843800	1.18305200	2.11303000
H	-2.93706000	0.21647600	3.58843100
H	-1.62079600	1.43402200	3.59263300
H	0.26945100	-1.84598000	3.33225100
H	0.07690800	-0.37065800	4.32268700
H	-1.21167300	-1.62024400	4.32046400
C	-1.53626800	3.06457000	-0.87593900
C	-0.57243100	3.63327200	-1.92966100
C	-1.47613600	3.86914200	0.43180700
H	-2.57123400	3.13326300	-1.27795600

H	-0.64503600	3.06482500	-2.87370400
H	-0.80595000	4.69195600	-2.14912000
H	0.46642800	3.56932600	-1.56023200
H	-2.17866000	3.45791600	1.17765800
H	-0.45465100	3.82451800	0.85142900
H	-1.73526100	4.93059200	0.25992900
O	-2.11390200	-2.24926500	-0.67640000
C	-3.18974000	-1.67584600	-0.64330700
C	-3.79326700	-0.37839000	-1.16049700
H	-3.28958300	0.52070100	-0.75839200
H	-3.83739700	-0.30722700	-2.25927300
C	-5.07231500	-0.87937000	-0.45139200
H	-5.87543700	-1.19903800	-1.13653000
O	-4.32753600	-2.11587900	-0.01547800
C	-5.60438800	-0.10602200	0.73695000
H	-6.11191400	0.81068200	0.38663600
H	-6.33533900	-0.70896300	1.30258300
H	-4.78642300	0.18702300	1.41685600

Free energy : -1.933.009950 hartrees

$TS_{0\text{-}1BL}$

Imaginary Frequency at: - 151 cm^{-1}

C	2.69510100	2.15778300	0.66181100
C	3.94533600	2.58759900	0.20021200
C	4.94145900	1.64483000	-0.09251000
C	4.66568800	0.28160300	0.07587000
C	3.40527000	-0.17294200	0.51895500
C	2.40483700	0.78765600	0.82450300
N	1.07447500	0.42928100	1.29294600
S	3.15105800	-1.94553200	0.70804700
C	2.69914800	-2.44699400	-1.01672800
C	1.56875000	-1.61646000	-1.65212800
C	1.23706700	-2.19612700	-3.04128700
O	0.42079100	-1.61919600	-0.83027600
Al	-0.37342200	-0.12000600	-0.28506200
H	1.91887200	2.89725100	0.89463300
H	4.13948000	3.65915700	0.08234100
H	5.92787500	1.96690000	-0.44307200
H	5.43482200	-0.46821700	-0.13742400
H	3.60486700	-2.42469800	-1.64977900
H	2.38115800	-3.49975900	-0.89809200
H	1.94977800	-0.58354800	-1.80384800
H	0.48860800	-1.55335500	-3.53446200
H	2.13071600	-2.24727500	-3.69136300
H	0.81525100	-3.21324000	-2.94150300
H	0.58518100	1.32107800	1.46118100
C	1.05939100	-0.28860500	2.65246700
C	0.04901700	-1.43523600	2.66553100

C	0.81903100	0.74419800	3.76247700
H	2.06614900	-0.71835800	2.77175700
H	0.26760300	-2.13623700	1.84338000
H	0.11714400	-1.97998800	3.62440800
H	-0.98339000	-1.06522000	2.55025600
H	1.58551500	1.54022700	3.74397700
H	-0.17734400	1.21059600	3.65166600
H	0.85929900	0.25952800	4.75378400
O	-2.06087900	-0.83008900	0.05174600
O	-0.02115500	1.04843500	-1.54189600
C	-0.45157900	2.36524800	-1.84706300
C	-1.70811900	2.32760000	-2.73304600
C	0.69595500	3.11125400	-2.54780200
H	-0.70968100	2.91173700	-0.91151900
H	-2.54135000	1.83847800	-2.20108600
H	-2.03062900	3.34779700	-3.01560200
H	-1.49717900	1.75967800	-3.65777800
H	1.59969100	3.12148700	-1.91407400
H	0.95323600	2.60375200	-3.49556900
H	0.41401700	4.15595200	-2.77702200
C	-2.79774100	-1.90069400	-0.56071200
C	-2.25065600	-3.25687200	-0.09395400
C	-2.81820400	-1.76479900	-2.08812900
H	-3.83911100	-1.79716100	-0.18329800
H	-2.28770000	-3.33379200	1.00721500
H	-2.84700200	-4.08485000	-0.52005300
H	-1.20160400	-3.35625200	-0.41843700
H	-3.22141900	-0.78218800	-2.38621700
H	-1.79108800	-1.85756500	-2.47959800
H	-3.44259400	-2.55475000	-2.54531000
O	-1.43245900	1.21845700	0.97124500
C	-2.63112200	0.82097300	0.91412400
C	-3.64298100	0.40947700	1.99056500
H	-3.79221200	-0.67557600	2.09282500
H	-3.45419500	0.86405400	2.97645600
C	-4.66626500	1.13592700	1.08400000
H	-4.99133600	2.10884500	1.49382100
O	-3.58226500	1.41454900	0.09519900
C	-5.83436300	0.37644200	0.48863300
H	-6.60309600	0.20400000	1.26385500
H	-6.29668900	0.95691600	-0.32837200
H	-5.51779600	-0.60018500	0.08548800

Free energy : -1932.978375 hartrees

***int1**$_{BL}$*

C	2.94929400	-1.97203000	0.83432800
C	4.17867600	-1.89213500	1.50107900
C	5.07454300	-0.85894800	1.19048400
C	4.72512900	0.07900500	0.20986600

C	3.48452600	0.02606900	-0.46004500
C	2.57835700	-1.01999900	-0.13633200
N	1.27098300	-1.14469400	-0.76221300
S	3.15697000	1.22926400	-1.75902000
C	2.50991600	2.68797300	-0.82235100
C	1.35397300	2.38802600	0.14851800
C	0.87185400	3.70844100	0.77953300
O	0.27788000	1.74640000	-0.51421500
Al	-0.29488300	0.17070500	0.04600000
H	2.25322100	-2.78426600	1.07806600
H	4.43568300	-2.64584600	2.25317700
H	6.04301100	-0.78979500	1.69727500
H	5.42152500	0.87916200	-0.06235500
H	3.34422100	3.17859000	-0.28863400
H	2.16518900	3.36462100	-1.62663700
H	1.75418400	1.75111100	0.96673900
H	0.08660700	3.49379500	1.52404800
H	1.69323500	4.24563100	1.28931600
H	0.44587900	4.37090400	0.00368100
H	0.83066100	-1.97581200	-0.33525400
C	1.32894300	-1.46000500	-2.27273500
C	0.38196300	-0.58379200	-3.09585600
C	1.06936500	-2.95933500	-2.47418000
H	2.36383700	-1.23027200	-2.57401500
H	0.53213700	0.47930300	-2.84695400
H	0.59915600	-0.72685900	-4.16992400
H	-0.66838700	-0.85586600	-2.90825600
H	1.79921900	-3.57762400	-1.91969700
H	0.04888100	-3.22113800	-2.13893400
H	1.15140800	-3.22110100	-3.54380900
O	-2.33320900	0.76960800	-0.22432700
O	0.03177600	0.01902900	1.75872400
C	-0.04740300	-1.06096300	2.67632100
C	-1.46626100	-1.20898300	3.24857800
C	0.98334800	-0.82855200	3.79213000
H	0.20855500	-2.02345600	2.16749300
H	-2.19867100	-1.39297600	2.44465600
H	-1.51622100	-2.04920800	3.96703500
H	-1.75283900	-0.28348400	3.78136300
H	1.99792700	-0.73160100	3.36939000
H	0.74482800	0.10758400	4.32924000
H	0.98092600	-1.65943600	4.52203900
C	-3.35952800	1.73420500	0.17627000
C	-3.09402000	3.01944100	-0.60786100
C	-3.32932600	1.91438800	1.69362400
H	-4.33019600	1.29565900	-0.12094500
H	-3.19240100	2.84656700	-1.69327800
H	-3.80930400	3.80684900	-0.31013100
H	-2.06744800	3.37052000	-0.41164900
H	-3.52479000	0.95387100	2.19552800
H	-2.34211600	2.28499200	2.01902800

H	-4.09906900	2.64375300	2.00544600
O	-1.40635100	-1.14940900	-0.62494000
C	-2.64749900	-0.60966700	-0.56087700
C	-3.64518100	-0.89419200	-1.71426300
H	-4.17530900	-0.00042000	-2.08373600
H	-3.21460100	-1.45284000	-2.55894300
C	-4.43671900	-1.69859700	-0.65966200
H	-4.30201800	-2.79257800	-0.76802000
O	-3.55142200	-1.20351500	0.40993000
C	-5.89815000	-1.36601300	-0.40919700
H	-6.05491400	-0.27492500	-0.33774400
H	-6.52638400	-1.75514800	-1.23143300
H	-6.24473600	-1.82573500	0.53302800

Free energy : -1932.986457 hartrees

$int2_{BL}$

C	-2.04962000	-2.13630600	-1.10701800
C	-3.01343800	-2.32950700	-2.10676800
C	-4.17698600	-1.54517500	-2.13547700
C	-4.35855000	-0.54589300	-1.16903200
C	-3.40005600	-0.34039200	-0.15783400
C	-2.24471800	-1.15943900	-0.11447200
N	-1.25737500	-0.94757900	0.92512300
S	-3.69802500	0.91002500	1.09693800
C	-2.48845600	2.28343800	0.67476100
C	-1.88816200	2.24765900	-0.74944400
C	-1.35564100	3.65117300	-1.10733500
O	-0.87996500	1.28241900	-0.90831800
Al	0.25832400	0.37153600	0.07913500
H	-1.12838700	-2.72552500	-1.10536700
H	-2.84731000	-3.09738900	-2.87055100
H	-4.93123900	-1.69748700	-2.91493300
H	-5.24741400	0.09310300	-1.18539700
H	-3.08302100	3.20287000	0.81789900
H	-1.67238000	2.27373300	1.41814900
H	-2.71163100	2.00800200	-1.45826600
H	-0.90329400	3.62553200	-2.11380600
H	-2.16403200	4.40542900	-1.11050200
H	-0.57754300	3.97204900	-0.39104700
O	0.74838000	0.95390200	1.66889300
C	1.76855900	1.88883100	2.01992000
C	2.64071100	1.29409200	3.13774100
C	1.13369800	3.21524200	2.46680700
H	2.41736000	2.08820600	1.14083500
H	3.10815900	0.34904300	2.80829300
H	3.44702200	1.99185900	3.43047600
H	2.02573600	1.07954300	4.03104800
H	0.52733500	3.65165700	1.65346700
H	0.47205500	3.04723100	3.33670400

H	1.90518800	3.95388300	2.75398400
C	3.65960200	-1.59234800	0.03848400
C	5.09349300	-1.09263600	0.21555300
C	3.52849500	-3.11316000	0.14068200
H	2.99453100	-1.10680000	0.77457400
H	5.13493900	0.00597500	0.13057900
H	5.48178300	-1.38319500	1.20803500
H	5.75427100	-1.52846700	-0.55498700
H	2.48092400	-3.41796300	-0.01466100
H	4.15552600	-3.60548100	-0.62423400
H	3.85534200	-3.46584200	1.13567600
O	1.00054000	-1.14985100	-0.67968500
C	1.98894000	-0.62577500	-1.42612300
C	1.73486300	-0.16546900	-2.88454700
C	1.80389300	1.30492400	-2.42001100
H	0.74775900	-0.48750500	-3.24873100
H	2.52606100	-0.47795500	-3.58419400
H	0.85791400	1.85643000	-2.53789000
O	1.94787200	0.93111100	-0.98667000
O	3.23825700	-1.17522300	-1.30279000
H	-1.71874100	-0.33727100	1.63100400
C	-0.80337300	-2.21064400	1.64575400
H	-0.15015300	-2.73800200	0.93020600
C	-1.98594400	-3.10857000	2.04751000
H	-2.57925600	-3.44516300	1.18217800
H	-1.60407000	-4.00448100	2.56828100
H	-2.66038700	-2.57672800	2.74388100
C	0.01973900	-1.82576300	2.88018100
H	0.82364400	-1.11876900	2.62878800
H	-0.62163300	-1.34482300	3.64266500
H	0.45019500	-2.73737200	3.33026000
C	3.00565300	2.11213600	-2.88254100
H	3.93902200	1.54130400	-2.73518000
H	2.91001700	2.35750000	-3.95655500
H	3.08064300	3.05818600	-2.31849400

Free energy : -1933.001585 hartrees

TS $_{2\text{-}3BL}$

Imaginary Frequency at: - 167 cm^{-1}

C	-1.91105300	-2.36698600	-0.71985100
C	-2.80982800	-2.79278000	-1.70848600
C	-3.98595800	-2.07001300	-1.95825000
C	-4.24704400	-0.90531400	-1.22243700
C	-3.35638500	-0.46806300	-0.22314100
C	-2.18153000	-1.21620100	0.04223800
N	-1.25316000	-0.75933700	1.05206400
S	-3.79969800	0.97091400	0.75637000
C	-2.55362100	2.28557400	0.28606400

C	-1.89642400	2.14121000	-1.10452800
C	-1.27557800	3.49631200	-1.50479800
O	-0.93581600	1.11456800	-1.15125500
Al	0.27634300	0.49508700	-0.02763500
H	-0.98364600	-2.91971800	-0.54629900
H	-2.58294500	-3.69504100	-2.28748100
H	-4.68970400	-2.40031600	-2.72982400
H	-5.14909600	-0.31487200	-1.41322600
H	-3.14340500	3.21821000	0.34068600
H	-1.76099600	2.32708800	1.05451700
H	-2.69546300	1.89783000	-1.83895100
H	-0.76709400	3.39454800	-2.47899700
H	-2.04303600	4.28753000	-1.59433800
H	-0.52533300	3.81783100	-0.75884700
O	0.61997800	1.34736500	1.48190900
C	1.46355500	2.48822900	1.65031400
C	2.90431000	2.05451500	1.96073900
C	0.88396300	3.36172700	2.77308000
H	1.48825600	3.09089700	0.71468400
H	3.30620900	1.46284500	1.12093700
H	3.56322500	2.92982500	2.11394700
H	2.92907400	1.43883600	2.87937500
H	-0.14402700	3.68281100	2.52868500
H	0.84643500	2.79024600	3.71852300
H	1.49935300	4.26577300	2.93581700
C	3.63745400	-1.70005100	0.36457800
C	5.10149900	-1.29480300	0.51497300
C	3.39434200	-3.19687100	0.56126400
H	2.99967500	-1.11452300	1.04800400
H	5.22817400	-0.21224300	0.34891300
H	5.45574600	-1.53556300	1.53263200
H	5.73355500	-1.83810900	-0.20956600
H	2.32931500	-3.43905300	0.41600700
H	3.99303200	-3.78447800	-0.15735000
H	3.68606700	-3.49972000	1.58286300
O	1.04251000	-1.19684600	-0.40921700
C	2.01506100	-0.87623300	-1.23616000
C	1.76228600	-0.57271100	-2.72655800
C	1.80427600	0.95843500	-2.52699700
H	0.77751900	-0.96150000	-3.03090800
H	2.54905700	-0.98539700	-3.37942200
H	0.86854600	1.45784900	-2.82969600
O	1.89102500	0.94175900	-1.06198900
O	3.25955900	-1.33936500	-1.01543500
H	-1.76095000	-0.04568600	1.61038500
C	-0.77316400	-1.82639600	2.02362000
H	-0.05760700	-2.44464800	1.45485800
C	-1.92557200	-2.70099100	2.54777700
H	-2.45186100	-3.23673000	1.74152100
H	-1.52736100	-3.45043500	3.25492600
H	-2.66466600	-2.08400200	3.09151900

C	-0.03495300	-1.15964700	3.19130200
H	0.72703300	-0.44719700	2.84045600
H	-0.74394100	-0.59949400	3.83015100
H	0.43650500	-1.93709300	3.81775300
C	3.02325400	1.65737600	-3.11583800
H	3.95283400	1.15158400	-2.79992700
H	2.97871000	1.65283900	-4.22078200
H	3.06626300	2.70610800	-2.77418800

Free energy : -1933.001585 hartrees

int3$_{BL}$

C	-0.34671400	-2.93591400	0.16061700
C	-0.71331700	-4.02912500	-0.63721000
C	-1.97297800	-4.06933800	-1.25093500
C	-2.85632800	-2.99646900	-1.06407800
C	-2.50257700	-1.89083500	-0.26682700
C	-1.22979000	-1.85814400	0.36687400
N	-0.84747000	-0.72494300	1.16907600
S	-3.72984700	-0.61217700	0.02573300
C	-3.23519200	0.81572800	-1.08589900
C	-2.13750600	0.52200300	-2.12848200
C	-2.07097500	1.69366800	-3.13094100
O	-0.87878400	0.28672800	-1.54617200
Al	-0.04501900	0.99668700	-0.15165600
H	0.64341800	-2.92060300	0.62541800
H	-0.00517800	-4.85434400	-0.77535300
H	-2.26533100	-4.92247200	-1.87244100
H	-3.84552600	-3.00164900	-1.53326500
H	-4.17348800	1.11087100	-1.58794700
H	-2.89738600	1.63140900	-0.42464000
H	-2.43187500	-0.39354800	-2.68828600
H	-1.26376900	1.50355300	-3.85942300
H	-3.01958300	1.81565900	-3.68622000
H	-1.84253600	2.64043600	-2.60851000
O	-0.96097400	1.94594800	1.04174300
C	-0.95243100	3.37244100	1.14889200
C	0.22529100	3.85225000	2.01199600
C	-2.29842500	3.82209400	1.73770600
H	-0.83506300	3.82869100	0.14205200
H	1.17660100	3.53189000	1.55561900
H	0.23409000	4.95554000	2.09563900
H	0.15516700	3.43123000	3.03226700
H	-3.13822500	3.49170500	1.10096700
H	-2.44267400	3.38326700	2.74244600
H	-2.34689200	4.92301000	1.82945400
C	3.99714000	-1.44727000	0.45167700
C	5.37467000	-0.94675400	0.87733800
C	3.95016200	-2.94146900	0.13635000

H	3.23243400	-1.18003900	1.19844100
H	5.35984500	0.13873700	1.07082700
H	5.68559200	-1.45945900	1.80431300
H	6.12693300	-1.15518300	0.09683600
H	2.94510100	-3.24247500	-0.20428000
H	4.67841100	-3.19721300	-0.65290200
H	4.19916800	-3.52440700	1.04045500
O	1.57793300	-0.23634900	-0.02840600
C	2.45341700	-0.17134900	-0.93289000
C	2.22854100	0.59007700	-2.18980600
C	1.98584600	2.11919200	-1.82011200
H	1.28610600	0.21817200	-2.63184400
H	3.06631100	0.46557500	-2.89351900
H	1.56244600	2.55555300	-2.75447200
O	1.09645200	2.26309100	-0.76103800
O	3.66220700	-0.72104100	-0.79694600
H	-1.71142800	-0.18238800	1.37243300
C	-0.19010500	-1.07347400	2.48333300
H	0.68097300	-1.70294600	2.23186400
C	-1.14526500	-1.87475400	3.38551200
H	-1.51380600	-2.78377900	2.88072100
H	-0.63064800	-2.18126600	4.31372100
H	-2.01805300	-1.25885200	3.67041800
C	0.33073600	0.17942900	3.19458000
H	1.07497500	0.71568000	2.58390300
H	-0.48552100	0.88544400	3.41488000
H	0.80944800	-0.11945800	4.14476000
C	3.31296700	2.83322400	-1.51283300
H	4.00448900	2.80546600	-2.37536600
H	3.10543000	3.88740100	-1.26300800
H	3.81745800	2.37501100	-0.64231900

Free energy : -1933.024551 hartrees
